# From Bugs to Bioplastics: Total (+)‐Dihydrocarvide Biosynthesis by Engineered *Escherichia coli*

**DOI:** 10.1002/cbic.201800606

**Published:** 2019-01-21

**Authors:** Gabriel A. Ascue Avalos, Helen S. Toogood, Shirley Tait, Hanan L. Messiha, Nigel S. Scrutton

**Affiliations:** ^1^ School of Chemistry Faculty of Science and Engineering University of Manchester 131 Princess Street Manchester M1 7DN UK

**Keywords:** (+)-dihydrocarvide monomer, Baeyer–Villiger monooxygenases, bioplastics, engineering, synthetic biology

## Abstract

The monoterpenoid lactone derivative (+)‐dihydrocarvide ((+)‐DHCD) can be polymerised to form shape‐memory polymers. Synthetic biology routes from simple, inexpensive carbon sources are an attractive, alternative route over chemical synthesis from (*R*)‐carvone. We have demonstrated a proof‐of‐principle in vivo approach for the complete biosynthesis of (+)‐DHCD from glucose in *Escherichia coli* (6.6 mg L^−1^). The pathway is based on the *Mentha spicata* route to (*R*)‐carvone, with the addition of an ′ene′‐reductase and Baeyer–Villiger cyclohexanone monooxygenase. Co‐expression with a limonene synthesis pathway enzyme enables complete biocatalytic production within one microbial chassis. (+)‐DHCD was successfully produced by screening multiple homologues of the pathway genes, combined with expression optimisation by selective promoter and/or ribosomal binding‐site screening. This study demonstrates the potential application of synthetic biology approaches in the development of truly sustainable and renewable bioplastic monomers.

## Graphical abstract

**Sustainable, renewable bioplastic monomers**: The monoterpenoid (+)‐ DHCD can be polymerised to form shape‐memory polymers. We have demonstrated a proof‐of‐principle approach to the complete biosynthesis of (+)‐ DHCD from glucose in *E. coli*, based on a modified *M. spicata* biosynthetic pathway. This route from a simple, inexpensive carbon source is more attractive than the alternative chemical synthesis from (*R*)‐carvone.

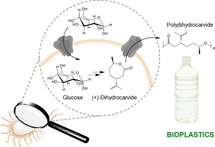

## Introduction

The increasing popularity of non‐petroleum‐based biopolymer applications is driven by concerns over dwindling fossil fuel supplies and the environmental impact of accumulating non‐biodegradable plastic waste.^1^ Industrially relevant biodegradable polymers include elastomers, resins and composites, which are often composed of cyclic ester monomers (lactones).^2^ For example poly‐ϵ‐caprolactone^3^ and polylactide^4^ are employed in drug delivery and tissue engineering,^5^ and are often major components in polyurethane biopolymers.^6^ A variety of limonene‐based monoterpenoids found in *Mentha* essential oils^7^ can be converted into the lactone monomers menthide, carvomenthide and (+)‐dihydrocarvide ((+)‐DHCD).^8^ Subsequent polymeric forms have uses as thermoplastic elastomers (shape‐memory polymers)^9^ and pressure‐sensitive adhesive components.^8^, ^10^

**Scheme 1:**
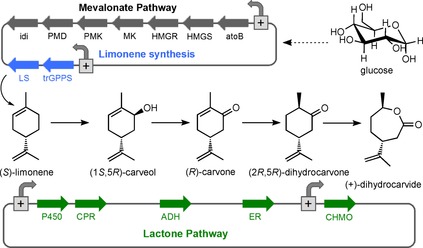
Synthetic biology approach to in vivo (+)‐dihydrocarvide monomer biosynthesis in *E. coli*. Enzymes: atoB=acetoacetyl‐CoA synthase, HMGS=hydroxymethylglutaryl‐CoA synthase, HMGR=hydroxymethylglutaryl‐CoA reductase, MK=mevalonate kinase, PMK=phosphomevalonate kinase, PMD=phosphomevalonate decarboxylase, idi=isopentyl diphosphate isomerase, trGPPS=N‐terminally truncated geranyl pyrophosphate synthetase, LS=limonene synthase, P450=limonene‐6‐hydroxylase, CPR=cytochrome P450 reductase, ADH=alcohol dehydrogenase, ER=ene‐reductase and CHMO=cyclohexanone monooxygenase.

Synthetic routes to monomeric (+)‐DHCD include hydrogenation and subsequent Baeyer–Villiger oxidation of the natural product (*R*)‐carvone.5a, 11 However, a synthetic biology route could serve as an alternative approach (Scheme 1) given that the enzymes responsible for (*R*)‐carvone biosynthesis in *Mentha spicata* are known,^7^ and prior studies with Baeyer–Villiger cyclohexanone monooxygenases (CHMO) have demonstrated (+)‐DHCD production.^8^, ^12^ An early attempt at in vivo (*R*)‐carvone production was to incorporate the C5 isoprenoid precursor (geranyl pyrophosphate) production and subsequent spearmint pathway genes into recombinant *Escherichia coli*.^13^ However this approach was unsuccessful owing to severe limitations in limonene precursor availability. This problem was overcome by feeding the cultures with (*S*)‐limonene, but production was limited by precursor uptake and cytotoxicity issues.^13^ A more recent study used orange peel as the limonene feedstock in mixed culture of two recombinant microorganisms containing the pathway genes for (1*S*,5*R*)‐carveol and (+)‐DHCD lactone biosynthesis, respectively.12d This was successful in generating (+)‐DHCD from waste orange peel; however, limonene cytotoxicity impacted on the upper level of feedstock concentrations that could be applied.12d To overcome this cytotoxicity, another study designed a cell‐free system for (*R*)‐carvone production from glucose.^14^ However, due to time constraints only limonene was efficiently produced.

We propose a more direct route, in which a *M. spicata*‐like pathway to (*R*)‐carvone production is combined with specific alcohol dehydrogenase (ADH) and CHMO enzymes within one recombinant strain of *E. coli* (Scheme 1). Limitations in C5 isoprenoid precursor production would be minimised by incorporating a second construct containing a eukaryotic mevalonate pathway to enable lactone production from simple carbon sources.^15^ The latter pathway was shown previously to substantially increase the in vivo production of the limonene derivative perillyl alcohol in *E*. *coli*.15a Homologues and modifications of key enzymes were screened in in vivo reactions to develop an optimised pathway to (+)‐DHCD. Functional pathway constructs underwent further modifications of the controlling elements (e.g., promoters) to enable significant levels of the terminal lactone product to be generated.

## Results and Discussion

### Limonene hydroxylation

The entry step into the *M*. *spicata* biosynthesis of (*R*)‐carvone is the hydroxylation of (*S*)‐limonene to (1*S*,5*R*)‐carveol (Scheme 1) catalysed by the cytochrome P450 enzyme limonene‐6‐hydroxylase (L6H) with its electron‐transfer partner cytochrome P450 reductase (CPR).^16^ Based on earlier studies, we generated an N‐terminally truncated and modified form of L6H^17^ (L6H_m_) to eliminate the signal sequence and increase soluble expression in *E*. *coli*. Unfortunately only a partial sequence was available for mint CPR (205 aa; GenBank: AW255332) from studies with expressed sequence tags (EST) from mint glandular trichomes.^18^ However the CPR from *Salvia miltiorrhiza* (Chinese sage; SmCPR) has high amino acid sequence homology (92 %) to the EST CPR sequence from mint.

Additionally, early studies with native L6H showed that hydroxylation occurs in the presence of a CPR from *Arabidopsis thaliana* (AtCPR).^17^ Therefore, we generated C‐terminally His_6_‐tagged versions of both SmCPR and AtCPR to determine the best electron‐transfer partner for L6H_m_.

**Figure 1:**
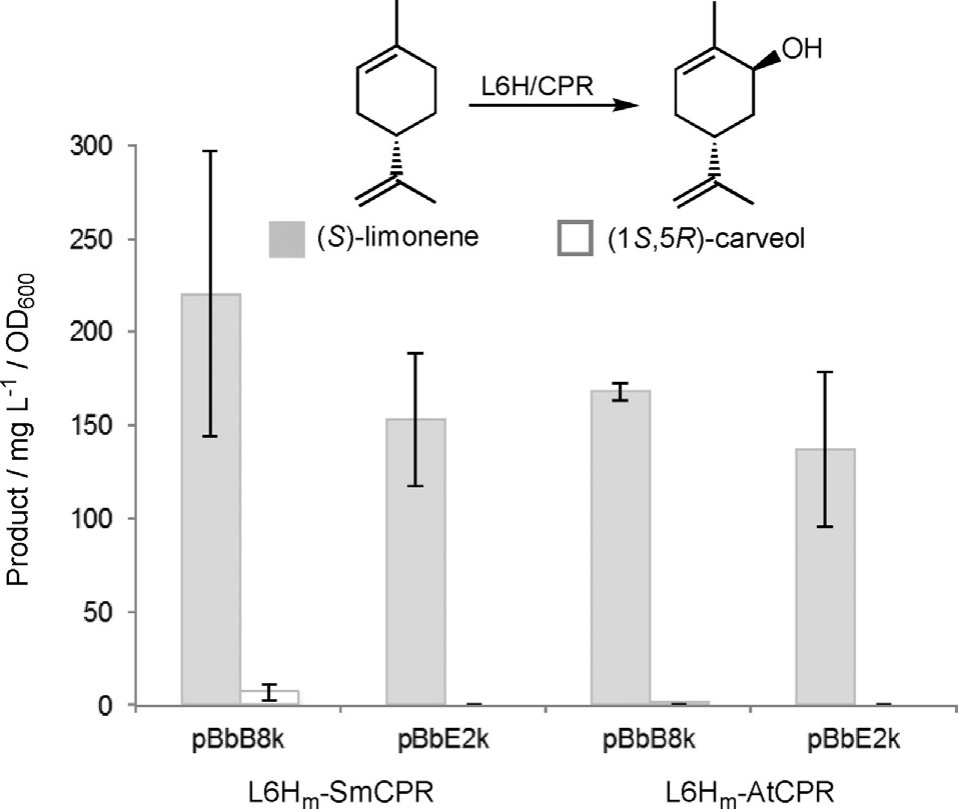
In vivo production of (*S*)‐limonene and (1*S*,5*R*)‐carveol by L6H_m_‐CPR constructs co‐expressed with limonene synthesis plasmid pJE16 410 in *E. coli*. Cultures (5 mL) were grown in Terrific broth, containing phosphate salts (9.4 g L^−1^ KH_2_PO_4_ and 2.2 g L^−1^ K_2_HPO_4_), 0.7 % (*w*/*v*) glucose, 60 μg mL^−1^ kanamycin and 100 μg mL^−1^ ampicillin. The culture was incubated at 37 °C at 200 rpm until the OD_600_ reached 0.4, then 25 μm IPTG (pJE16410), 500 μm δ‐aminolevulinic acid, 25 μm arabinose (pBbB8k) or 100 nm tetracycline (pBbE2k) was added. The cultures were incubated at 30 °C for 72 h. Each culture aliquot (1 mL) was cooled for 10 min on ice, then the nonane layer was extracted with ethyl acetate (2×375 μL) containing 0.01 % *sec*‐butyl benzene. Product yields and identification were determined by GC‐MS analysis. An OD_600_ of 1.0 corresponds to ≈1.7 g L^−1^ wet weight of cells.^19^ No detectable limonene hydroxylase activity was detected in control *E. coli* cells.

Initial co‐expression constructs of L6H_m_ with either SmCPR or AtCPR were generated in plasmid pCWori under the control of a *tac* promoter.^17^ In vitro biotransformations of cell lysates with limonene showed only poor (1*S*,5*R*)‐carveol production by L6H_m_ with either SmCPR or AtCPR over 24 h (e.g., (95.2±3.3) μm with SmCPR; 1.9 % yield; Figure S6 in the Supporting Information). Therefore new L6H_m_/CPR constructs were generated without His_6_ tags and controlled by either *araBAD* (arabinose) or *tet* (tetracycline) promoters on different plasmid backbones (pBbB8k and pBbE2k, respectively). We performed in vivo reactions for the detection of functional L6H_m_‐CPR pairs instead of using purified proteins or cell lysates. This is due to difficulties in obtaining sufficient quantities of soluble, active membrane‐associated L6H_m_ (results not shown). This method involved the co‐expression of L6H_m_‐CPR constructs with a limonene production plasmid pJBEI6410,15a thereby eliminating the need to supplement the culture with limonene. Cultures were grown in the presence or absence of a nonane overlay, which efficiently sequestered the monoterpenoids away from the aqueous phase to minimise cytotoxicity.

(*S*)‐Limonene production was detected in all cultures, with a range of titres of 137–220 mg L^−1^/OD_600_ dependent on the L6H_m_‐CPR construct (Figure 1). These differences likely reflect the efficiency of production versus the rate of utilisation by the expressed L6H_m_/CPR; however, the nature of the L6H_m_‐CPR plasmid backbone appeared to have an impact on limonene titres. The best (1*S*,5*R*)‐carveol‐producing construct was L6H_m_‐SmCPR in pBbB8k ((6.7±4.3) mg L^−1^/OD_600_), with the equivalent AtCPR‐containing plasmid showing a 20‐fold reduction in yield (Figure 1). The higher than expected variability in (1*S*,5*R*)‐carveol yields within replicates is a reflection on the nonoptimised growth and induction conditions; however, a clear preference for the sage CPR was seen. No detectable levels of (1*S*,5*R*)‐carveol were found with the constructs in the tetracycline‐inducible pBbE2k plasmid. This could be indirectly related to the higher copy number and promoter strength, leading to changes in soluble recombinant protein expression levels and/or a higher metabolic burden on *E*. *coli*. This was seen by an increase in the relative proportion of insoluble protein expressed in these constructs (results not shown).

Optimisation trials were performed in vivo with the best performing construct L6H_m_‐SmCPR in pBbB8k co‐expressed with the limonene production plasmid pJBEI6410. The presence/ absence of a *n*‐nonane bilayer, culture density at induction, inducer concentration (isopropyl‐β‐d‐thiogalactopyranoside (IPTG) and arabinose) and post‐induction time were varied (Table S10). In contrast to studies with in vivo production of limonene and other monoterpenoids,^20^ the presence of a *n*‐nonane co‐solvent reduced the levels of (1*S*,5*R*)‐carveol production at least sevenfold ((1.7±0.9) vs. (12.8±4.4) mg L^−1^/OD_600_). This is likely due to the sequestering of the (*S*)‐limonene generated by the pJBEI6410 plasmid into the co‐solvent, thereby reducing the intracellular concentrations and availability for the hydroxylation enzyme. Increasing the kanamycin concentration (selective for L6H_m_‐SmCPR) from 15 to 60 μg mL^−1^ led to a threefold increase in (1*S*,5*R*)‐carveol. The conditions leading to the highest yields of (1*S*,5*R*)‐carveol with the highest reproducibility were found to be induction at a mid‐log phase, with 25 μm IPTG and 25 mm arabinose ((33.8±5.0) mg L^−1^/OD_600_).

### Alcohol dehydrogenase selection

The second step in the *M. spicata* biosynthetic pathway is the NAD^+^‐dependent oxidation of (1*S*,5*R*)‐carveol to (*R*)‐carvone (Scheme 1) catalysed by (−)‐isopiperitenol/(−)‐carveol dehydrogenase (IPDH).^7^ This ADH is a member of the zinc‐dependent short‐chain dehydrogenase/reductase superfamily similar to human 17β‐hydroxysteroid dehydrogenase.^21^ We performed in vitro biotransformations of cell lysates of IPDH expressed in *E*. *coli* strain BL21(DE3) with a (1*S*,5*R*)‐ and (1*R*,5*R*)‐carveol mix. Unfortunately only minor (*R*)‐carvone yields were obtained, only about two times higher than that obtained by constitutive *E*. *coli* ADHs alone ((0.33±0.17) vs. (0.14±0.01) μm). Therefore, we cloned three additional IPDH homologues to identify the best performing enzyme capable of generating (*R*)‐carvone in *E*. *coli*.

The first homologue was (1*S*,5*R*)‐carveol dehydrogenase from *Rhodococcus erythropolis* DCL14 (CDH),^21^ known to oxidise each of the four isomers of carveol, but with the highest affinity and turnover rate with the desired (1*S*,5*R*)‐carveol. The second candidate was the ADH from *Rhodococcus ruber* DSM 44541 (RrADH), which is specific for a variety of *S* secondary alcohols, such as cyclohexanol.^22^ The final homologue screened was the ADH from *Lactobacillus kefir* DSM 20587 (LkADH).^23^ This enzyme differed by being NADP^+^‐dependent, *R*‐selective, and required Mg^2+^ for activity. Prior studies with cell extracts of *E*. *coli* expressing LK‐ADH showed a 21 % conversion of a mixture of (1*S*,5*R*)‐ and (1*R*,5*R*)‐carveol stereoisomers.^24^

**Figure 2:**
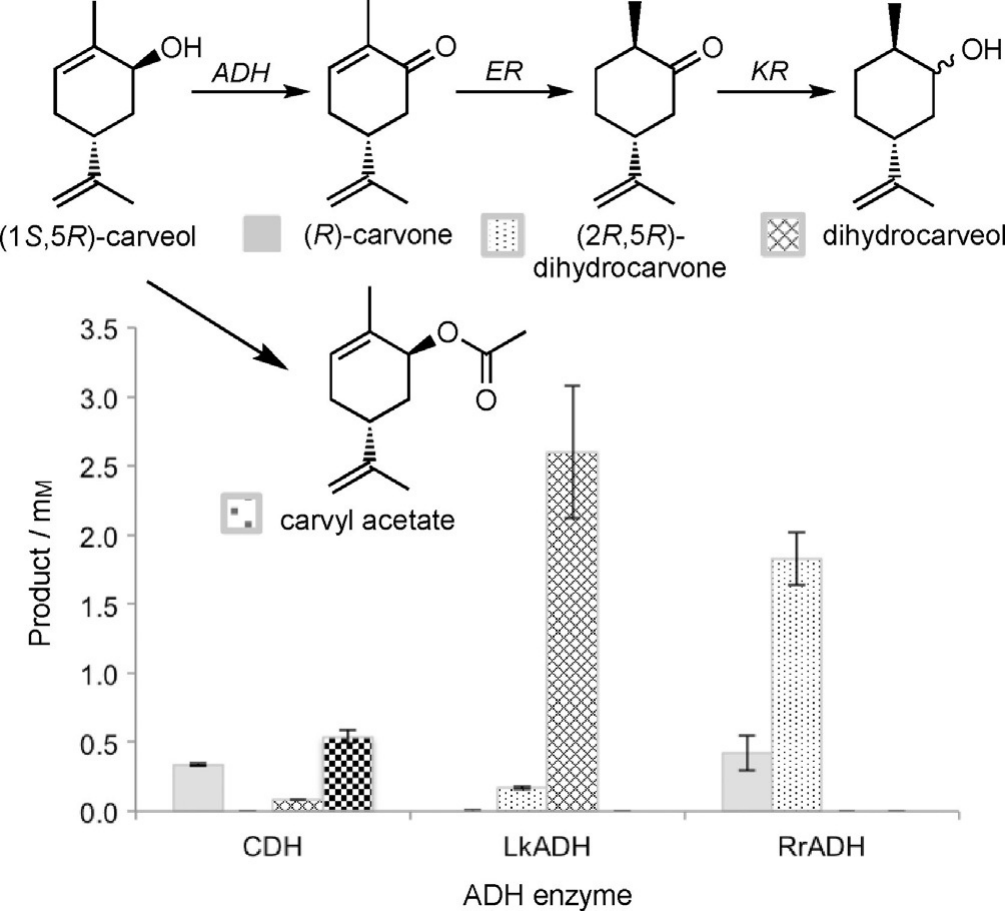
In vitro biotransformations of ADH enzymes in *E. coli* cell extracts, showing the proposed pathway for the formation of by‐products (2*R*,5*R*)dihydrocarvone, dihydrocarveol isomers and carvyl acetate. The dihydrocarvone produced is ≈90 % 2*R*,5*R* isomer. No detectable native *E. coli* ADH activity with carveol was detected during in vivo reactions (Figure 1). ER: ene‐reductase, KR: ketoreductase.

Each gene was expressed in *E. coli* strain BL21(DE3), and clarified cell lysates were used for in vitro biotransformations with the (1*S*,5*R*)‐ and (1*R*,5*R*)‐carveol mix. Unfortunately, *E. coli* contains constitutive Old Yellow Enzymes (OYEs) and ketoreductases, which are likely to consecutively produce (2*R*,5*R*)‐dihydrocarvone^25^ and dihydrocarveol isomers, respectively from (*R*)‐carvone (Figure 2). Therefore evidence of each recombinant ADH activity above control cell lysates is apparent from the detection of one or more of three potential products.

Cell lysates of RrADH showed the highest yields of (*R*)‐carvone ((0.42±0.13) mm), closely followed by CDH ((0.34±10) mm; Figure 2). However, higher levels of by‐product dihydrocarvone isomers ((1.83±0.19) mm) and neo‐dihydrocarveol ((2.6±0.5) mm) were detected with RrADH and LkADH, thus suggesting high activity of the earlier ADH step. An additional by‐product carvyl acetate ((0.54±0.05) mm) was seen in reactions with CDH lysate, presumably generated by the action of an *E*. *coli* alcohol acetyltransferase on carveol.^26^ Therefore, potentially each of these ADH enzymes could be used to catalyse in vivo (1*S*,5*R*)‐carveol dehydrogenation within *E*. *coli*.

### (*S*)‐Limonene to (*R*)‐carvone operons

The next stage involved combining the highest performing (1*S*,5*R*)‐carveol‐producing construct (L6H_m_‐SmCPR in pBbB8k) with the four ADH enzymes to find the optimal set of biocatalysts. Each operon was constructed by inserting the ADH gene downstream from SmCPR, separated by one of two ribosome binding sequences. RBS1 (GAATA ACTAT TTAAG AGGGA GATTA ATAAC A) has a predicted translation rate of 13 969,^27^ whereas RBS2 (TAAGGAGGT) was chosen as it successfully increased the production of *p*‐coumaryl alcohol in *E*. *coli* when using a tricistronic operon.^28^ Each construct was co‐transformed with plasmid pJBEI6410 into *E*. *coli* strain NEB10β to screen for the in vivo production of (*R*)‐carvone from glucose.

**Figure 3:**
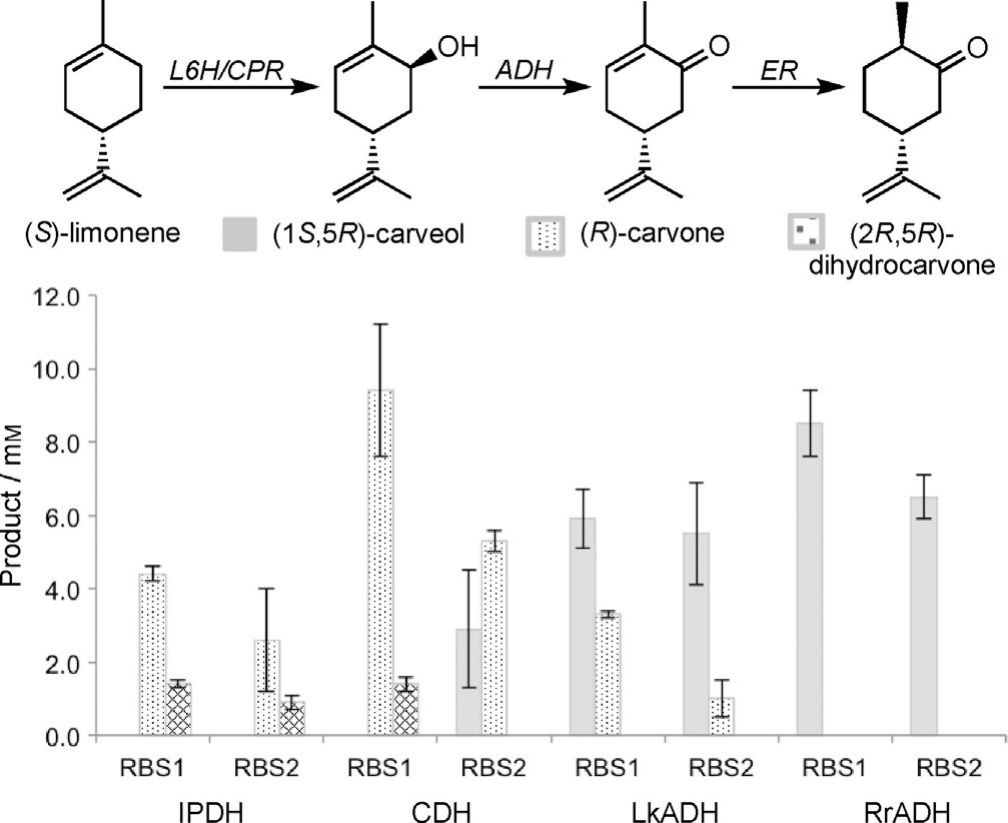
In vivo production of (*R*)‐carvone and other monoterpenoids by L6H_m_‐SmCPR‐ADH constructs in *E. coli*, coexpressed with limonene synthesis plasmid pJE16410, showing the proposed pathway for product formation. Cultures (5 mL) were grown in Terrific broth containing phosphate salts (9.4 g L^−1^ KH_2_PO_4_ and 2.2 g L^−1^ K_2_HPO_4_), 0.7 % (*w*/*v*) glucose, 60 μg mL^−1^ kanamycin and 100 μg mL^−1^ ampicillin. The culture was incubated at 37 °C and 200 rpm until the OD_600_ reached 0.4, then 25 μm IPTG (pJE16410), 500 μm δ‐aminolevulinic acid and 25 μm arabinose were added. Cultures were incubated at 30 °C for 72 h. Culture aliquots (3 mL) were extracted with ethyl acetate (2×375 μL) containing 0.01 % *sec*‐butyl benzene. Product yields and identification were determined by GC‐MS analysis.

Constructs containing CDH showed the highest levels of (*R*)‐carvone production ((71±10) mg L^−1^ with RBS1). In contrast, IPDH‐containing constructs showed a twofold reduction in yield (36.5±1.8 with RBS1; Figure 3). In both cases, (2*R*,5*R*)dihydrocarvone was present due to the action of an *E*. *coli* ene‐reductase. RrADH cultures only showed the presence of (1*S*,5*R*)‐carveol, thus suggesting a lack of functional ADH protein expression. LkADH cultures also contained significant levels of (1*S*,5*R*)‐carveol, with only moderate ADH activity detected (Figure 3). Therefore, CDH was chosen as the biocatalyst for the in vivo production of (*R*)‐carvone in *E*. *coli*.

### Biocatalyst selection and screening for (2*R*,5*R*)‐dihydrocarvone and (+)‐DHCD production

The NADPH‐dependent C=C reduction of (*R*)‐carvone to (2*R*,5*R*)‐dihydrocarvone is a well‐known reaction catalysed by OYE family members.^25^, ^29^ We selected the classical OYE subclass member pentaerythritol tetranitrate reductase (PETNR) from *Enterobacter cloacae* PB2 as the biocatalyst for this step, as it is highly expressed in *E*. *coli* and is known to react with (*R*)‐carvone to produce (2*R*,5*R*)‐dihydrocarvone with high yields and diastereoselectivity (94 % *de*).^30^

For the next step, the flavin‐dependent cyclohexanone monooxygenases (CHMO) catalyse the NADPH‐dependent Baeyer–Villiger oxidation of cyclic ketones to form cyclic esters (lactones).^31^ The CHMO from *Rhodococcus* species Phi1 (CHMO_WT_) catalyses the oxidation of (2*R*,5*R*)‐dihydrocarvone; however, it generates the unwanted abnormal lactone (3*S*,6*S*)‐6‐isopropenyl‐3‐methyl‐2‐oxo‐oxepanone.^32^ Site‐directed mutagenesis studies of this enzyme generated a triple variant (F249A/F280A/F435A; CHMO_3M_) that successfully produced the required “normal” (+)‐DHCD lactone.^8^ Therefore, we selected CHMO_3M_ as the catalyst for the final lactone production step in *E*. *coli*.

To assess the performance of these two enzymes in *E. coli*, a variety of multigene constructs were generated and assessed for both (2*R*,5*R*)‐dihydrocarvone and (+)‐DHCD production under standard fermentation conditions. Cell extracts of each construct were tested by in vitro biotransformations in the presence of a commercially available (1*S*,5*S*)‐ and (1*R*,5*R*)‐carveol mix, NAD^+^ (IPDH) and an NADPH cofactor‐recycling system (PETNR and CHMO_3M_). These early constructs contained the complete pathway from (*S*)‐limonene to (+)‐DHCD (L6H_m_‐IPDH‐PETNR‐CHMO_3M_; L6H_m_IPC_3M_) except for CPR, as the most suitable CPR (and ADH homologue) had not been determined at the time of pathway construction. However, the focus of these operon designs was to generate the most suitable PETNR‐CHMO_3M_ gene arrangement to maximise (+)‐DHCD production from exogenously supplied (*R*)‐carvone, so the absence of CPR and the presence of IPDH instead of CDH was inconsequential. Full details of the production of these constructs can be found in the Supporting Information (Experimental Sections 1–5, Tables S1–S7 and Figures S1–S4).

PETNR is known to be highly expressed and active in *E*. *coli* extracts,^33^ so the main focus of the multiple L6H_m_IPC_3M_ designs was to increase the expression of CHMO_3M_. An initial construct was generated with the genes under control of a single *lac*UV5 promoter (L6H_m_IPC_3M_). Biotransformations with carveol showed the production of (*R*)‐carvone ((0.87±0.03) mm) and dihydrocarvone ((0.34±0.03) mm) above control *E*. *coli* extracts, the latter predominantly the 2*R*,5*R* enantiomer (Table S11). No (+)‐DHCD was detected, probably due to the low levels of the (2*S*,5*R*)‐dihydrocarvone present. To further check for CHMO_3M_ expression, biotransformations were performed with (*R*)‐carvone, thereby eliminating the need for the IPDH step. This generated both (2*S*,5*R*)‐dihydrocarvone ((1.47±0.08) mm) and (+)‐DHCD ((0.11±0.01) mm), thus suggesting the presence of active CHMO_3M_. However, higher expression levels of CHMO_3M_ are required to enable efficient (+)‐DHCD production from earlier pathway intermediates. Three additional L6H_m_IPC_3M_ constructs were generated in which the ribosomal binding‐site sequence upstream of CHMO_3M_ was varied in an attempt to increase its expression levels. However, no (+)‐DHCD was detected during biotransformations, even in the presence of CHMO_3M_ substrate (2*S*,5*R*)‐dihydrocarvone (Table S12).

The next approach to boost expression was to insert a variety of promoters upstream of CHMO_3M_. The selected promoters were induced by IPTG (*trc*/*lacO*, *tacII*/*lacO*, *lac*UV5), rhamnose (*rha*BAD) or tetracycline (*PtetA*), allowing either a single (IPTG) control over the expression of all three genes or differential control for CHMO_3M_.^34^ Biotransformations of cell extracts were performed with three different substrates to determine the most effective expression control system for CHMO_3M_ (Tables 1, S13 and S14). As expected, in each case, the highest (+)‐DHCD production was seen in the presence of (2*S*,5*R*)‐dihydrocarvone (CHMO_3M_ substrate), with the best yields obtained with CHMO_3M_ under the control of a *trc*/*lacO* promoter ((0.57±0.07) mm; Table 1). When the CHMO_3M_ promoter was substituted for *PtetA* and *rha*BAD, the yields decreased by 1.7‐ and 4.4‐fold, respectively. Biotransformations in the presence of carveol showed a significant decrease in (+)‐DHCD production ((0.12±0.01) mm with *trc*/*lacO*). In the case of the *rha*BAD‐containing construct, no (+)‐DHCD was produced in the presence of carveol. Therefore, the inclusion of the promoters *trc*/*lacO* and *PtetA* upstream of CHMO_3M_ have successfully led to the production of (+)‐DHCD from carveol.

**Table 1:** In vitro monoterpenoid production by L6HIPpC_3M_ constructs with three different promoters upstream of CHMO_3M_.^[a]^

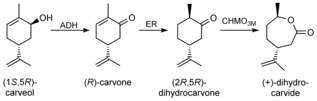				
CHMO_3M_	Substrate	(2*R*,5*R*)‐DHC	(+)‐DHCD	DHCL
promoter		[mm]	[mm]	[mm]
*trc*/*lacO* ^[a]^	carveol	0.05±0.01	0.12±0.01	0.04±0.01
	carvone	2.36±0.13	0.47±0.06	trace
	DHC mix	–	0.57±0.07	trace
*PtetA*	carveol	0.11±0.01	0.05±0.02	0.03±0.01
	carvone	0.92±0.15	0.28±0.05	n.d.
	DHC mix	–	0.33±0.06	n.d.
*rha*BAD	carveol	0.15±0.01	n.d.	0.41±0.07
	carvone	2.29±0.07	0.11±0.02	0.10±0.02
	DHC mix	–	0.13±0.03	n.d.

[a] No C‐His_6_ tag on PETNR. Reactions (1 mL) were performed in buffer (50 mm Tris, pH 7.0) containing cell lysate, 5 mm (*R*)‐carvone, 150 μm NAD^+^, ±15 μm NADP^+^, 15 mm glucose and 10 U GDH. Reaction mixtures were incubated for 24 h at 30 °C and 130 rpm. Monoterpenoids were extracted with 2×0.5 mL ethyl acetate containing 0.1 % *sec*‐butylbenzene internal standard. Product yields and identification were determined by GC‐MS analysis using a DB‐WAX column. No evidence was seen of native *E. coli* lactone formation. DHC mix: (2*R*,5*R*)‐ and (2*S*,5*R*)‐dihydrocarvone, DHCD: dihydrocarvide lactone, DHCL: dihydrocarveol by‐product; trace: ≤0.02 mm, n.d.: none detected. The data for the production of (*R*)‐carvone and the by‐product carvyl acetate are given in Tables S13 and S14.

### Lactone production from glucose

Full pathway assembly was performed by using the most successful carvone‐producing construct as the backbone (L6H_m_‐SmCPR‐CDH RBS1; arabinose inducible), and inserting PETNR‐promoter‐CHMO_3M_ genes downstream of CDH. Constructs L6HIP‐*trc*‐C_3M_ and L6HIP‐*tet*‐C_3M_ were chosen as the source of PETNR‐promoter‐CHMO_3M_ genes due to their ability to produce (+)‐DHCD in the presence of carveol. Additionally, the L6HIP‐*rha*‐C_3M_ construct was chosen as it generated significant (+)‐DHCD in the presence of (*R*)‐carvone. The three dual promoter constructs (L6HCCP‐*trc*‐C_3M_, L6HCCP‐*tet*‐C_3M_ and L6HCCP‐*rha*‐C_3M_) were co‐expressed in *E*. *coli* with the limonene synthesis plasmid for total in vivo production of (+)‐DHCD lactone from glucose. Given the length of the number of steps in the pathway to (+)‐DHCD, CHMO_3M_ inducer was added either at the same time as the other inducers (IPTG and arabinose) or 6 h later so as to give time for the intermediate monoterpenoid concentrations to build up within the cell. The *trc*‐promoter is IPTG inducible, so the expression of CHMO_3M_ in construct L6HCCP‐*trc*‐C_3M_ could not be postponed for 6 hours, as IPTG is required for the induction of the limonene synthesis genes (pJBEI6410 plasmid).

**Figure 4:**
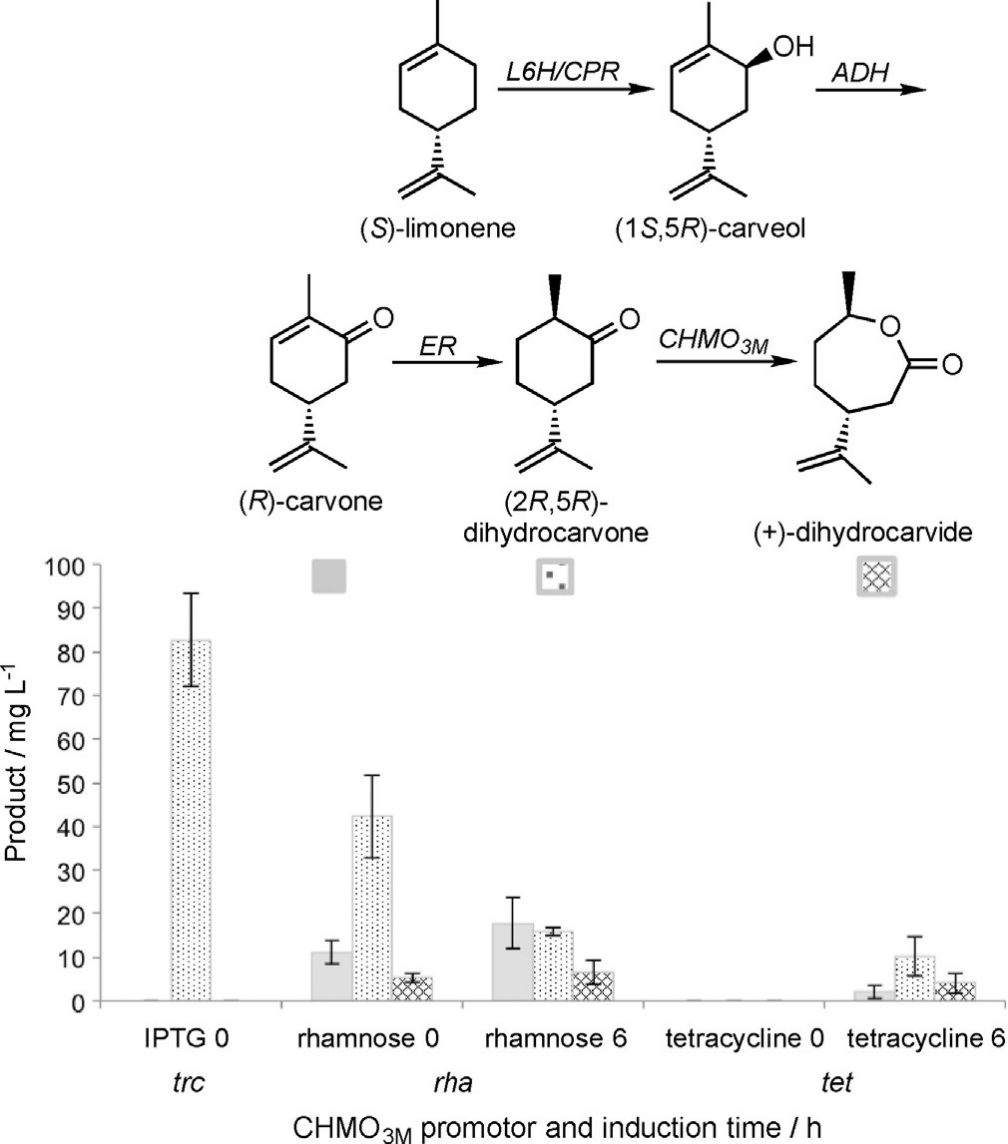
In vivo monoterpenoid production from limonene by L6CCPpC_3M_ constructs with three different promoters upstream of CHMO_3M_, co‐expressed with limonene synthesis plasmid pJE16410. Cultures (5 mL) were grown in Terrific broth containing phosphate salts (9.4 g L^−1^ KH_2_PO_4_ and 2.2 g L^−1^ K_2_HPO_4_), 0.7 % (*w*/*v*) glucose, 60 μg mL^−1^ kanamycin and 100 μg mL^−1^ ampicillin. The culture was incubated at 37 °C and 200 rpm until the OD_600_ reached 0.4, then 25 μm IPTG (pJE16410), 500 μm δ‐aminolevulinic acid, 25 μm arabinose and either 0.05 % rhamnose or 100 nm tetracycline were added at the time of induction or after 6 h. Cultures were incubated at 30 °C for 72 h. Aliquots (3 mL) were extracted with ethyl acetate (2×375 μL) containing 0.01 % *sec*‐butyl benzene. Product yields and identification were determined by GC‐MS analysis.

In vivo studies showed that two of the three construct combinations successfully generated (+)‐DHCD from glucose (Figure 4). The most successful limonene to lactone‐producing construct in *E*. *coli* was L6HCCP‐*rha*‐C_3M_, which showed around 6 mg L^−1^ (+)‐DHCD, dependent on the induction conditions. Interesting, the highest in vitro (+)‐DHCD‐producing construct L6HCCP‐*trc*‐C_3M_ did not show any detectable levels of (+)‐DHCD under in vivo conditions when co‐expressed with the limonene‐producing plasmid. This highlights the importance of screening multiple constructs with different controlling elements, as the addition of an extra IPTG‐inducible pathway can sometimes have an (unpredictable) impact on the expression of each recombinant gene.

## Conclusions

In vivo production of fine chemicals is one possible solution to the increasing demand for sustainable and renewable manufacturing. The cost‐effectiveness of biological manufacturing strategies is dependent on the construction of recombinant microorganisms that express the correct “assembly line” of enzymes at sufficient levels. We have achieved a proof‐of‐principle demonstration of in vivo production of the bioplastics precursor (+)‐DHCD in *E*. *coli*, grown on a simple, inexpensive carbon source. This overcomes the severe limitations in the existing partial pathway approach (limonene to lactone) caused by the addition of a cytotoxic precursor (limonene) supply to the microorganism.^13^ The in vivo production of limonene in *E*. *coli* overcomes the precursor uptake constraints, and minimises cytotoxicity by the effective removal of the precursor molecules by the remaining pathway steps.

Further studies are required to increase the productivity and cost‐effectiveness of this bio‐manufacturing approach to bioplastics production. This is necessary to increase the production titres, concomitant with the elimination of selection agents (antibiotics) and expensive chemical induction (e.g., IPTG and rhamnose). For example, host selection and (chromosomal) modification could be applied to reduce the cytotoxicity and recovery of the monoterpenoids and increase cellular export. A high‐throughput combinatorial approach could be applied to screen for the best combination of enzyme homologues/variants, vector backbone, promoter combination and gene order. However our demonstration of the complete in vivo production of (+)‐DHCD is a leap forward in the development of truly sustainable and renewable bioplastic monomers.

## Experimental Section

**General materials and reagents**: All reagents and solvents were purchased from commercial suppliers, and were of analytical grade or better. Media components were obtained from Formedium (Norfolk, UK). Gene sequencing and oligonucleotide syntheses were performed by Eurofins MWG (Ebersberg, Germany). The BglBrick series of vectors and the mevalonate pathway overexpression plasmid pJBEI641015a were obtained from Addgene (https://www.addgene.org).^34^

**Gene synthesis and subcloning**: The genes encoding the C‐terminally His_6_‐tagged proteins pentaerythritol tetranitrate reductase (PETNR C‐His_6_) from *E*. *cloacae* (UNIPROT: P71278)^35^ and cyclohexanone monooxygenase (CHMO_WT_) from *Rhodococcus* species Phi1 (UNIPROT: Q84H73)^8^ were synthesised and subcloned into pET21b, as described previously. The CHMO triple variant F249A/F280A/F414A (CHMO_3M_) was generated by PCR mutagenesis, as described previously.^8^ The following C‐terminally His_6_‐tagged alcohol dehydrogenase genes were synthesised and subcloned into pET21b by GenScript: (−)‐*trans*‐isopiperitenol dehydrogenase from *Mentha piperita* (IPDH; UNIPROT: Q5C919),^7^ (1*S*,5*R*)‐carveol dehydrogenase from *R*. *erythropolis* (CDH; UNIPROT: Q9RA05),^21^ (*R*)‐specific alcohol dehydrogenase from *L*. *kefir* (LkADH; UNIPROT: Q6WVP7)^36^ and secondary alcohol dehydrogenase from *R*. *ruber* DSM 44541 (RrADH; UNIPROT: Q8KLT9).^23^ Each gene was codon optimised for optimal expression in *E. coli*. In the case of IPDH, a stop codon was inserted before the XhoI site by overlap extension PCR^37^ to eliminate the C‐terminal His_6_ tag.

The gene encoding an N‐terminally modified mature (4*S*)‐limonene‐6‐hydroxylase from *M. spicata* (L6H_m_; UNIPROT: Q9XHE8)^17^ was synthesised and subcloned without codon optimisation into pCWori (+) by Geneart. The N terminus was modified by removing the chloroplast signal sequence in addition to other modifications designed to increase its soluble expression in *E*. *coli*, as described previously.^17^ Two C‐terminally His_6_‐tagged cytochrome P450 reductases from *A*. *thaliana* (AtCPR; UNIPROT: Q9SB48) and *S*. *miltiorrhiza* (SmCPR; UNIPROT: S4URU2) were synthesised and subcloned into pET21b by incorporating codon‐optimisation techniques of rare codon removal. Each gene was transformed into competent cells of *E*. *coli* strain BL21(DE3) for functional overexpression according to the manufacturers′ protocols.

**Limonene hydroxylation construct assembly**: Functional limonene hydroxylation constructs were generated by In‐Fusion cloning (Takara)^38^ between PCR linearised L6H_m_ (3′‐end) in pCWori (+) and either AtCPR or SmCPR, with the inclusion of a Shine–Dalgarno sequence between the genes (L6H_m_‐AtCPR and L6H_m_‐SmCPR, respectively). The constructs were transformed into *E*. *coli* strain JM109 for functional expression. The two constructs were further subcloned into vectors pBbB8k‐RFP and pBbE2k‐RFP (Addgene)^34^ under the control of tetracycline and pBAD promoters, respectively. This was performed by using In‐Fusion cloning between PCR linearised vector (RFP eliminated) and an L6H‐CPR insert. Following each PCR reaction, the template was removed by DpnI digestion, and PCR product size was determined by 0.6 % agarose gel electrophoresis. The oligonucleotide sequences encoding all the PCR primers can be found in Table S1. The correct assembly of each construct was confirmed by DNA sequencing. Each construct was co‐transformed with plasmid pJBEI6410 into competent cells of *E*. *coli* strain NEB10β for functional overexpression according to the manufacturers′ protocols.

**Production and in vitro biotransformations of ADH lysates**: ADH clones were grown in lysogeny broth (10 g L^−1^ tryptone, 5 g L^−1^ yeast extract and 5 g L^−1^ NaCl) containing ampicillin (100 μg mL^−1^) and starter culture (2 %). In the case of CDH production, sorbitol (182 g L^−1^) and betaine**⋅**HCl (0.293 g L^−1^) were included in the medium. Cultures were incubated at 37 °C until the OD_600 nm_ reached 0.5. Protein production was induced by the addition of IPTG (100 μm), followed by incubation at 30 °C for 16–18 h. Cells were harvested by centrifugation (4000 *g*), and the pellets were resuspended in lysis buffer (1.7 mL; 50 mm Tris, pH 7.0, containing EDTA‐free complete protease inhibitor cocktail, 1 mm MgCl_2_, 0.1 mg mL^−1^ DNase I, 0.1 mg mL^−1^ lysozyme and 10 % glycerol). Cell‐free supernatants were generated by sonication (10 cycles of 10 s on/1 min off at 40 % amplitude) and centrifugation for (5 min, 13 000 *g*). The presence of the individual recombinant proteins was determined by SDS‐PAGE on 12 % Mini‐PROTEAN‐TGX stain‐free gels (Bio‐Rad). Protein content was visualised by using a Safe Imager 2.0 Blue light trans‐illuminator (Bio‐Rad).

Reactions (1 mL) were performed in buffer (50 mm Tris pH 7.0) containing a mixture of (1*S*,5*R*)‐ and (1*R*,5*R*)‐carveol (5 mm), NADP^+^ (15 μm), glucose (15 mm) and glucose dehydrogenase (GDH; 10 U). Reaction mixtures were incubated for 72 h at 30 °C and 130 rpm. Control reactions were performed with *E*. *coli* lysates that did not contain the recombinant plasmids. In each case, monoterpenoids were extracted with ethyl acetate (2×0.5 mL) containing 0.1 % *sec*‐butylbenzene internal standard. Product yields and identification were determined by GC and GC‐MS analysis, respectively, on a DB‐WAX column.

**Generation of the L6H‐CPR‐ADH constructs**: Eight constructs were generated in which each ADH was inserted downstream of the CPR gene of L6H_m_‐SmCPR in pBbB8k preceded by one of two different ribosome binding sequences (*rbs1–2*). This was performed by using In‐Fusion cloning between PCR linearised L6H_m_‐SmCPR and amplified rbs‐ADH insert (L6H_m_‐SmCPR‐IPDH, L6H_m_‐SmCPR‐CDH, L6H_m_‐SmCPR‐LkADH and L6H_m_‐SmCPR‐RRADH versions 1 and 2, respectively). Following each PCR, template removal and DNA clean up were performed as above. The oligonucleotide sequences encoding the PCR primers can be found in Table S2. The correct assembly of each construct was confirmed by DNA sequencing. Each construct was cotransformed with plasmid pJBEI6410 into competent cells of *E*. *coli* strain NEB10β for functional overexpression according to the manufacturers′ protocols.

**Construction of multienzyme‐cascade constructs containing PETNR and CHMO_3M_**: A series of multigene constructs containing L6H_m_, IPDH, PETNR and CHMO_WT_ or CHMO_3M_ were generated to maximise the production of (+)‐DHCD lactone from (1*S*,5*R*)‐carveol. The optimisation parameters varied were the plasmid backbone (pBbE1c or pBbE5c), RBS sequences and the presence of four different promoters upstream of CHMO_3M_. Full details of the assembly techniques and biotransformation data performed for each construct can be found in the Supporting Information.

**Construction of the complete lactone‐producing pathway from limonene**: (+)‐DHCD‐producing constructs from (*S*)‐limonene (Figure S5) were generated by In‐Fusion cloning between the PCR linearised L6H_M_‐SmCPR‐CDH construct (contains *rbs1*) in pBbB8k and one of three PETNR‐promoter‐CHMO_3M_ inserts amplified from L6HIP‐*tet*‐C_3M_, L6HIP‐*rha*‐C_3M_ and L6HIP‐*trc*‐C_3M_. These inserts differ by the type of promoter located upstream of the CHMO_3M_ gene, that is tetracycline‐, rhamnose‐ or IPTG‐inducible, respectively. PCR linearisation of L6H_M_‐SmCPR‐CDH was performed between the 3′‐end of CDH and the terminator region,; amplification of the PETNR‐*promoter*‐CHMO_3M_ inserts included *rbs2* upstream of PETNR. Following each PCR reaction, template removal and DNA clean up were performed as above. The oligonucleotide sequences encoding the PCR primers can be found in Table S8. The correct assembly of each construct was confirmed by DNA sequencing (L6HCCP‐*tet*‐C_3M_, L6HCCP‐*rha*‐C_3M_ and L6HCCP‐*trc*‐C_3M_). Each construct was cotransformed with plasmid pJBEI6410 into competent cells of *E*. *coli* strain NEB10β for functional overexpression according to the manufacturers′ protocols. A summary of all the gene constructs is found in Table S9.

**In vivo biotransformations**: A single colony of *E*. *coli* NEB10β cotransformed with pJBEI6410‐ and pBbB8k‐containing biosynthetic constructs was used to inoculate Terrific broth (5 mL) containing phosphate salts (9.4 g L^−1^ KH_2_PO_4_ and 2.2 g L^−1^ K_2_HPO_4_), glucose (0.7 %, *w*/*v*) kanamycin (60 μg mL^−1^) and ampicillin (100 μg mL^−1^). The culture was incubated at 37 °C and 200 rpm until the OD_600_ reached 0.4, then IPTG (25 μm), arabinose (25 mm), δ‐aminolevulinic acid (500 μm) were added with/without tetracycline (100 nm) and with/without rhamnose (0.05 %). The cultures were incubated at 30 °C for 72 h unless otherwise stated. Each culture aliquot (3 mL) was cooled for 10 min on ice, then extracted with ethyl acetate (2×375 μL) containing 0.01 % *sec*‐butyl benzene.^8^ Product yields and identification were determined by GC‐MS analysis.

**Analytical techniques**: Monoterpenoid content was quantified by using an Agilent Technologies 7890A GC system with a flame ionization detector (FID). Biotransformation extracts (1 μL) were analysed on a DB‐WAX column (30 m; 0.32 mm; 0.25 μm film thickness; JW Scientific). In this method, the injector temperature was 220 °C with a split ratio of 20:1. The carrier gas was helium with a flow rate of 1 mL min^−1^ and a pressure of 5.1 psi. The program began at 40 °C with a hold for 2 min, then the temperature was increased to 210 °C at a rate of 15 °C min^−1^, with a final hold at 210 °C for 3 min. The FID was maintained at a temperature of 250 °C with a flow of hydrogen at 30 mL min^−1^. Product was quantitated by comparing the peak areas to those of authenticated standards of known concentration. Where authentic standards were not commercially available (by‐products only), the concentrations were estimated by using an average concentration per peak area value based on 11 related monoterpenoid standards.

Monoterpenoids were identified on an Agilent Technologies 7890B GC system with a 5977A MSD extractor EI source detector by using the same DB‐WAX column. In this method, the injector temperature was 240 °C with a split ratio of 50:1. The carrier gas was helium with a flow rate of 3 mL min^−1^ and a pressure of 8.3 psi. The program began at 50 °C with a hold for 1 min, then the temperature was increased to 68 °C at a rate of 5 °C min^−1^, with a hold at 68 °C for 2 min. A second temperature gradient was applied at 25 °C min^−1^ until 230 °C with a final hold of 2 min. The mass spectra fragmentation patterns were entered into the NIST/EPA/NIH 11 mass spectral library to identify any potential match.

**Upscaled in vitro biotransformations and analysis**: Reactions (10 mL) were performed in buffer (50 mm Tris, pH 7.0) containing (*R*)‐carvone/(+)‐dihydrocarvone starting substrate (5 mm; ≈30 mg), NADP^+^ (10 μm), glucose (15 mm), GDH (10 U) and the enzyme(s) (10 μm). The samples were incubated for 24 h at 30 °C and 180 rpm, then cooled in ice, and the organic compound(s) were extracted with petroleum ether (PET; 1:2, *v*/*v*). Two further PET extractions were performed, and the pooled organic phase was dried over anhydrous MgSO_4_. The product(s) were recovered following solvent removal with a rotor evaporator with the water bath set to 30 °C, at 20–30 Torr. Product(s) was/were purified chromatographically on silica gel (pore size 60, 220–240 mesh size, particle size 35–75 μm), which was equilibrated with 100 % PET. The compounds were eluted with a mix of PET and ether (5–40 %), and each elution fraction was analysed by thin‐layer chromatography (TLC) using a mobile phase composed of a PET/ether (70:30). The TLC plate was stained with phosphomolybdic acid stain (PMA; 12 g in 250 mL ethanol) and exposed to UV light. The fractions containing the desirable metabolites were pooled, and the solvent was removed as before.

^1^H and ^13^C NMR spectra of the scaled‐up purified product(s) (10 mg mL^−1^) in deuterated chloroform were recorded on a Bruker Avance 400 MHz NMR spectrometer at 298 K without the addition of an internal standard. Chemical shifts were calibrated against the residual solvent signal. ^1^H and ^13^C NMR spectra were analysed by using MestreNova.

## Conflict of interest

*The authors declare no conflict of interest*.

## Supporting information

As a service to our authors and readers, this journal provides supporting information supplied by the authors. Such materials are peer reviewed and may be re‐organized for online delivery, but are not copy‐edited or typeset. Technical support issues arising from supporting information (other than missing files) should be addressed to the authors.

Supplementary

## References

[1a] A. Gandini , T. M. Lacerda , Prog. Polym. Sci. 2015, 48, 1–39;

[1b] S. A. Miller , ACS Macro Lett. 2013, 2, 550–554. 10.1021/mz400207g 35581816

[2] Y. Zhu , C. Romain , C. K. Williams , Nature 2016, 540, 354–362. 27974763 10.1038/nature21001

[3] M. Labet , W. Thielemans , Chem. Soc. Rev. 2009, 38, 3484–3504. 20449064 10.1039/b820162p

[4] A. P. Gupta , V. Kumar , Eur. Polym. J. 2007, 43, 4053–4074.

[5a] K. J. Zhu , X. Lin , S. Yang , J. Appl. Polym. Sci. 1990, 39, 1–9;

[5b] M. Vert , S. M. Li , G. Spenlehauer , P. Guerin , J. Mater. Sci. Mater. Med. 1992, 3, 432–446;

[5c] P. Mainil-Varlet , B. Rahn , S. Gogolewski , Biomaterials 1997, 18, 257–266. 9031728 10.1016/s0142-9612(96)00126-3

[6] S. A. Gurusamy-Thangavelu , S. J. Emond , A. Kulshrestha , M. A. Hillmyer , C. W. Macosko , W. B. Tolman , T. R. Hoye , Polym. Chem. 2012, 3, 2941–2948.

[7] K. L. Ringer , E. M. Davis , R. Croteau , Plant Physiol. 2005, 137, 863–872. 15734920 10.1104/pp.104.053298 PMC1065387

[8] H. L. Messiha , S. T. Ahmed , V. Karuppiah , R. Suardiaz , G. A. Avalos , N. Fey , S. Yeates , H. S. Toogood , A. J. Mulholland , N. S. Scrutton , Biochemistry 2018, 57, 1997–2008. 29533655 10.1021/acs.biochem.8b00169

[9] J. R. Lowe , W. B. Tolman , M. A. Hillmyer , Biomacromolecules 2009, 10, 2003–2008. 19505135 10.1021/bm900471a

[10a] M. J. L. Tschan , E. Brule , P. Haquette , C. M. Thomas , Polym. Chem. 2012, 3, 836–851;

[10b] M. A. Hillmyer , W. B. Tolman , Acc. Chem. Res. 2014, 47, 2390–2396. 24852135 10.1021/ar500121d

[11] D. Zhang , M. A. Hillmyer , W. B. Tolman , Biomacromolecules 2005, 6, 2091–2095. 16004449 10.1021/bm050076t

[12a] K. Balke , M. Bäumgen , U. T. Bornscheuer , ChemBioChem 2017, 18, 1627–1638; 28504873 10.1002/cbic.201700223

[12b] K. Balke , M. Kadow , H. Mallin , S. Saß , U. T. Bornscheuer , Org. Biomol. Chem. 2012, 10, 6249–6265; 22733152 10.1039/c2ob25704a

[12c] H. Leisch , K. Morley , P. C. K. Lau , Chem. Rev. 2011, 111, 4165–4222; 21542563 10.1021/cr1003437

[12d] N. Oberleitner , A. K. Ressmann , K. Bica , K. Gartner , P. Gartner , M. W. Fraaije , U. T. Bornscheuer , F. Rudroff , M. D. Mihovilovic , Green Chem. 2017, 19, 367–371.

[13] O. A. Carter , R. J. Peters , R. Croteau , Phytochemistry 2003, 64, 425–433. 12943759 10.1016/s0031-9422(03)00204-8

[14] A. Casini , F.-Y. Chang , R. Eluere , A. King , E. M. Young , Q. M. Dudley , A. Karim , K. Pratt , C. Bristol , A. Forget , A. Ghodasara , R. Warden-Rothman , R. Gan , A. Cristofaro , A. E. Borujeni , M.-H. Ryu , A.-T. J. Li , Y. C. Kwon , H. Wang , E. Tatsis , C. Rodriguez-Lopez , S. O'Connor , M. H. Medema , M. Fischbach , M. C. Jewett , C. A. Voigt , B. Gordon , J. Am. Chem. Soc. 2018, 140, 4302–4316. 29480720 10.1021/jacs.7b13292

[15a] J. Alonso-Gutierrez , R. Chan , T. S. Batth , P. D. Adams , J. D. Keasling , C. J. Petzold , T. S. Lee , Metab. Eng. 2013, 19, 33–41; 23727191 10.1016/j.ymben.2013.05.004

[15b] T. P. Korman , P. H. Opgenorth , J. U. Bowie , Nat. Commun. 2017, 8, 15526. 28537253 10.1038/ncomms15526 PMC5458089

[16] S. Lupien , F. Karp , M. Wildung , R. Croteau , Arch. Biochem. Biophys. 1999, 368, 181–192. 10415126 10.1006/abbi.1999.1298

[17] C. Haudenschild , M. Schalk , F. Karp , R. Croteau , Arch. Biochem. Biophys. 2000, 379, 127–136. 10864450 10.1006/abbi.2000.1864

[18] B. M. Lange , M. R. Wildung , E. J. Stauber , C. Sanchez , D. Pouchnik , R. Croteau , Proc. Natl. Acad. Sci. USA 2000, 97, 2934–2939. 10717007 10.1073/pnas.97.6.2934 PMC16033

[19] J. Glazyrina , E. M. Materne , T. Dreher , D. Storm , S. Junne , T. Adams , G. Greller , P. Neubauer , Microb. Cell Fact. 2010, 9, 42. 20509968 10.1186/1475-2859-9-42 PMC2891675

[20] N. G. H. Leferink , A. J. Jervis , Z. Zebec , H. S. Toogood , S. Hay , E. Takano , N. S. Scrutton , ChemistrySelect 2016, 1, 1893–1896. 29756025 10.1002/slct.201600563 PMC5947754

[21] M. J. van der Werf , C. van der Ven , F. Barbirato , M. H. Eppink , J. A. de Bont , W. J. van Berkel , J. Biol. Chem. 1999, 274, 26296–26304. 10473585 10.1074/jbc.274.37.26296

[22] C. Hamnevik , C. Blikstad , S. Norrehed , M. Widersten , J. Mol. Catal. B: Enzym. 2014, 99, 68–78.

[23] W. Hummel , A. Riebel , Ann. N. Y. Acad. Sci. 1996, 799, 713–716.

[24] N. Oberleitner , C. Peters , J. Muschiol , M. Kadow , S. Saß , T. Bayer , P. Schaaf , N. Iqbal , F. Rudroff , M. D. Mihovilovic , U. T. Bornscheuer , ChemCatChem 2013, 5, 3524–3528.

[25] H. S. Toogood , J. M. Gardiner , N. S. Scrutton , ChemCatChem 2010, 2, 892–914.

[26] A. Aharoni , L. C. P. Keizer , H. J. Bouwmeester , Z. Sun , M. Alvarez-Huerta , H. A. Verhoeven , J. Blaas , A. M. M. L. van Houwelingen , R. C. H. De Vos , H. van der Voet , R. C. Jansen , M. Guis , J. Mol , R. W. David , M. Schena , A. J. van Tunen , A. P. O'Connell , Plant Cell 2000, 12, 647–661. 10810141 10.1105/tpc.12.5.647 PMC139918

[27] H. M. Salis , E. A. Mirsky , C. A. Voigt , Nat. Biotechnol. 2009, 27, 946–950. 19801975 10.1038/nbt.1568 PMC2782888

[28] P. V. van Summeren-Wesenhagen , R. Voges , A. Dennig , S. Sokolowsky , S. Noack , U. Schwaneberg , J. Marienhagen , Microb. Cell Fact. 2015, 14, 79–88. 26062542 10.1186/s12934-015-0274-9 PMC4464236

[29] H. S. Toogood , D. Mansell , J. M. Gardiner , N. S. Scrutton in Comprehensive Chirality, 1st ed. (Eds.: H. Yamamoto, E. Carreira), Elsevier, Oxford, 2011, pp. 216–260.

[30] H. S. Toogood , A. Fryszkowska , M. Hulley , M. Sakuma , D. Mansell , G. M. Stephens , J. M. Gardiner , N. S. Scrutton , ChemBioChem 2011, 12, 738–749. 21374779 10.1002/cbic.201000662

[31] D. Sheng , D. P. Ballou , V. Massey , Biochemistry 2001, 40, 11156–11167. 11551214 10.1021/bi011153h

[32] P. C. Brzostowicz , D. M. Walters , S. M. Thomas , V. Nagarajan , P. E. Rouvière , Appl. Environ. Microbiol. 2003, 69, 334–342. 12514013 10.1128/AEM.69.1.334-342.2003 PMC152449

[33] H. S. Toogood , A. Ní Cheallaigh , S. Tait , D. J. Mansell , A. Jervis , A. Lygidakis , L. Humphreys , E. Takano , J. M. Gardiner , N. S. Scrutton , ACS Synth. Biol. 2015, 4, 1112–1123. 26017480 10.1021/acssynbio.5b00092

[34] T. S. Lee , R. A. Krupta , F. Zhang , M. Hajimorad , W. J. Holtz , N. Prasad , S. K. Lee , J. D. Keasling , J. Biol. Eng. 2011, 5, 12. 21933410 10.1186/1754-1611-5-12 PMC3189095

[35] M. E. Hulley , H. S. Toogood , A. Fryszkowska , D. Mansell , G. M. Stephens , J. M. Gardiner , N. S. Scrutton , ChemBioChem 2010, 11, 2433–2447. 21064170 10.1002/cbic.201000527

[36] W. Stampfer , B. Kosjek , C. Moitzi , W. Kroutil , K. Faber , Angew. Chem. Int. Ed. 2002, 41, 1014–1017; 10.1002/1521-3773(20020315)41:6<1014::aid-anie1014>3.0.co;2-6 12491297

[37] Y.-H. Xiao , M.-H. Yin , L. Hou , M. Luo , Y. Pei , Biotechnol. Lett. 2007, 29, 925–930. 17356793 10.1007/s10529-007-9327-4

[38] J. Park , A. L. Throop , J. LaBaer , Curr. Protoc. Mol. Biol. 2015, 110, 320.1–3.20.23. 25827088 10.1002/0471142727.mb0320s110 PMC4492480

