# Supplementary material for: From Bugs to Bioplastics: Total (+)‐Dihydrocarvide Biosynthesis by Engineered Escherichia coli
Source: Chembiochem. 2019 Jan 21;20(6):785–92. doi: 10.1002/cbic.201800606 (PMC6850611; doi:10.1002/cbic.201800606)
Supplement: Supplementary file 1 — Supplementary [file CBIC-20-785-s001.pdf]

CHEMBIOCHEM

Supporting Information

From Bugs to Bioplastics: Total (+)-Dihydrocarvide Biosynthesis by Engineered *Escherichia coli*

Gabriel A. Ascue Avalos, Helen S. Toogood, Shirley Tait, Hanan L. Messiha, and Nigel S. Scrutton*[a]

cbic_201800606_sm_miscellaneous_information.pdf

Supporting Information

Contents

Experimental

1. Oligonucleotide sequences for initial constructs assembly 2

2. Generation of L6H$_m$-IPDH-PETNR-CHMO$_{WT}$ constructs 4

3. Generation of L6H$_m$-IPDH-PETNR-CHMO$_{3M}$ construct version 1 5

4. Generation of L6H$_m$-IPDH-PETNR-CHMO$_{3M}$ constructs version 2 5

5. Generation of L6H$_m$-IPDH-PETNR-CHMO$_{3M}$ constructs version 3 6

6. *In vitro* biotransformation of L6H$_m$-IPDH-PETNR-CHMO$_{3M}$ constructs 7

7. Generation of a full lactone-producing pathway 8

8. Summary of constructs 9

Results and Discussion

1. *In vitro* biotransformations of cell extracts of L6H$_m$ co-expressed with SmCPR or AtCPR 10

2. *In vivo* biotransformations of the L6H$_M$-SmCPR construct 10

3. *In vitro* biotransformations of the L6HIPC$_{3M}$-E5c construct 11

4. *In vitro* biotransformations of the L6HIPC$_{3M}$ 2a-2c constructs 12

5. *In vitro* biotransformations of the seven L6HIPpC$_{3M}$ constructs 13

References 14

Experimental

1. Oligonucleotide sequences for initial constructs assembly

Table S1. Oligonucleotide sequences used for PCR amplifications.

Name	Sequence
IPDH stop codon insertion	
IPDHStF	CGTTTGTGGCGGTGGCTAAA*TGA*GATCCGGCTGCTAACAAAGCCC
IPDHStR	CTTTGTTAGCAGCCGGATC*TCA*TTTAGCCACCGCCACAAACGGCAG
L6H-AtCPR constructs generation	
L3HWEndF	TCTAGAGTCGACCTGCAGCCCAAGCTTATC
L6HWStR	ATTTAGCTG**TCCTCC**TCAAGGACTTTTATAGAGTGTGGGAACCAAGC
CPRAtFullF	**GGAGGA**CAGCTAAATGACCTCGGCTCTGTATGCGTCG
CPRAtFullR	CAGGTCGACTCTAGATCACCAAACATCGCGCAGATAACGGCCTTC
CPRSmFullF	**GGAGGA**CAGCTAAATGATGGAAAGCACCAGCGTTAAACTGAGTC
CPRSmFullR	CAGGTCGACTCTAGATCACCAAACGTCACGCAGATAACGACCG
L6H-CPR pairs in pBbB8k	
L6H(tr)_RBS_pBb_F	AATTCAAAAGATCTAGGAGGATAAAGAAATGGCTCTGTTATTAGCAGTTTTTTTGTC
CPR(At)_pBb_R	TACTCGAGTTTGGATCCTCACCAAACATCGCGCAGATAAC
CPR(Sm)_pBb_R*	ACTCGAGTTTGGATCCTCACCACACGTCACGCAGATAAC
pBbOpenNewF	GGATCCAAACTCGAGTAAGG
P450_lin_Rw	CCTAGATCTTTTGAATTCCCAA
L6H-CPR pairs in pBbE2k	
L6H(no)_RBSpCW_F	AGATCTTTTAAGAAGTTTCACACAGGAAACAGGATCCATCGATG
L6H(opt)RBS21bF	AGATCTTTTAAGAAGTTTGTTTAACTTTAAGAAGGAG
CPR(At)TmL_pBbR	CTCGAGTTTGGATCCTCACCACACATCGCGCAG
CPR(Sm)TmL_pBbR	CTCGAGTTTGGATCCTCACCACACGTCACGCAG
pBbOpenNewF	GGATCCAAACTCGAGTAAGG
pBbOpenR	CTTCTTAAAAGATCTTTTGAATTC

Mutations are shown in italics. New Shine-Dalgarno sequences upstream of CPR are shown in bold. All sequences are 5’ to 3’. *Single nucleotide synonymous mutation is marked as an underlined nucleotide.

Table S2. Oligonucleotide sequences used for the construction of L6H-CPR-ADH combinations in pBbB8k.

Name	Sequence
PCR amplification of parts for L6H-CPR-ADH in pBbB8k with rbs1 upstream of ADH	
pBbB8k_F	TGAGGATCCAAACTCGAGTAAGGA
CPR_R	TCACCAAACGTCACGCAGATAAC
RBS1-CPR(Sm)*F	TCTGCGTGACGTTTGGTGA**GAATAACTATTTAAGAGGGAGATTAATAAC**
IPDH-pBbB8k*R	TCCTTACTCGAGTTTGGATCCTCATTTAGCCACCGCCACAA
CDH_rbs1CPRSm*F	CTGCGTGACGTTTGGTGA**GAATAACTATTTAAGAGGGAGATTAATAAC**AATGGCTCGTGTGGAAGGTCA
CDH_bb*R	CTTACTCGAGTTTGGATCCTCATTTCAGGGTCGTGCCAG
LK-RBS1-CPRSm_F	CTGCGTGACGTTTGGTGA**GAATAACTATTTAAGAGGGAGATTAATAAC**AATGACCGATCGTCTGAAAGGTA
LK_pBbb8K_R	GAGTTTGGATCCTCACTCGAGCTGTGCGGTATAACC
RR-rbs1-CPRSm_F	CTGCGTGACGTTTGGTGA**GAATAACTATTTAAGAGGGAGATTAATAAC**AATGAAAGCACTGCAGTATACCGA
PDHStR	GAGTTTGGATCCTCACTCGAGTCCCGGAACAACAA
PCR amplification of parts for L6H-CPR-ADH in pBbB8k with rbs2 upstream of ADH	
pBbB8k_F	TGAGGATCCAAACTCGAGTAAGGA
CPR_R	TCACCAAACGTCACGCAGATAAC
IPDH-rbs2CPRSM*F	TCTGCGTGACGTTTGGTGA**TAAGGAGGT**ATACCATGGCATCTGTGAAAAAACTGGC
IPDH-pBbB8k*R	TCCTTACTCGAGTTTGGATCCTCATTTAGCCACCGCCACAA
CPRSmFullF	GGAGGACAGCTAAATGATGGAAAGCACCAGCGTTAAACTGAGTC
CDH_rbs2CPRSm*F	CTGCGTGACGTTTGGTGA**TAAGGAGGT**ATACCATGGCTCGTGTGGAAGGTCA
CDH_bb*R	CTTACTCGAGTTTGGATCCTCATTTCAGGGTCGTGCCAG
LK-rbs2-CPR(Sm)_F	CTGCGTGACGTTTGGTGA**TAAGGAGGT**ATACCATGACCGATCGTCTGAAAGGTA
LK_pBbb8K_R	GAGTTTGGATCCTCACTCGAGCTGTGCGGTATAACC
RR-rbs2-CPRSm_F	CTGCGTGACGTTTGGTGA**TAAGGAGGT**ATACCATGAAAGCACTGCAGTATACCGA
RR_pBbb8K_R	GAGTTTGGATCCTCACTCGAGTCCCGGAACAACAA

The rbs sequences are shown in bold. All sequences are 5’ to 3’.

2. Generation of $L6H_m$-IPDH-PETNR-$CHMO_{WT}$ constructs

The generation of the initial lactone operon versions was performed prior to the determination of the optimal $L6H_m$-CPR pair and ADH homologue. Therefore no CPR gene was included at this stage, and IPDH was the chosen ADH. The initial construct ($L6HIPC_{WT}$), containing $L6H_m$, IPDH, PETNR-His_8 and $CHMO_{WT}$, was assembled into vectors pBbE1c and pBbE5c[1] under the control of the promoters pTrc and TrcLacUV5prom, respectively ($L6HIPC_{WT}$-E1c and $L6HIPC_{WT}$-E5c; Figure S1). Both the vectors and each gene insert were linearised and amplified, respectively, by PCR, including the insertion of unique RBS sequences between successive genes. Spacer sequences were incorporated at the 3' end of PETNR-His_8 and $CHMO_{WT}$-His_6 to eliminate potential assembly problems due to the repetitive 18 bp His-tag sequences. In some cases, the primers incorporated silent mutations into the genes to eliminate oligo hairpin formation that would inhibit PCR amplification. Following each PCR reaction, template removal was performed by DpnI digestion, and PCR product size was determined by 0.6% agarose gel electrophoresis. The oligonucleotide sequences encoding the PCR primers can be found in Supporting Information Table S3.

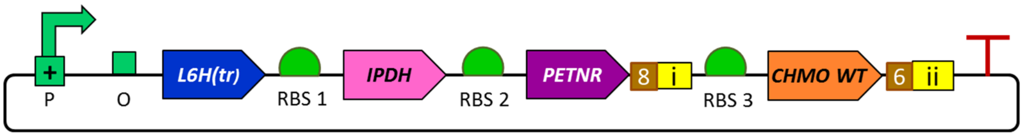

Figure S1. Design of the $L6HIPC_{WT}$ construct. The yellow boxes indicate the position of the 3' spacer sequences. The IPTG inducible promoter (P) and operator (O) are shown as green arrows and boxes, respectively. The different RBS sequences are shown as green semicircles, with the terminator indicated by a red T. The numbers in brown boxes indicate the number of histidine residues in the tag. L6H(tr) = $L6H_m$.

Construct assembly was performed via the ligase cyclase reaction methodology described previously,[2] using bridging oligos (Table S3) to ensure the correct order of the genes. The correct assembly of each construct was confirmed by DNA sequencing. Each construct was transformed into competent cells of *E. coli* strain NEB10β for functional overexpression according to the manufacturers protocols.

Table S3. Oligonucleotide sequences used for the construction of $L6HIPC_{WT}$-E1c and $L6HIPC_{WT}$-E5c.

Name	Sequence
PCR amplification of parts for $L6HIPC_{WT}$-E1c and $L6HIPC_{WT}$-E5c	
L6HOpF	GCAGTTTTTTTGTCGGCAATTATAATCCTTGTGG
L6H_RBSOpR	TTAAATAGTTATTCTCACGGACTTTTATAGAGTGTGGGAACCAAGC
IPDH-RBBOpF	GAGGGAGATTAATAACAATGGCATCTGTGAAAAAACTGGCTGG
IPDH-RBBOpR	TCCTAGAGTGCGAATTTCTCATTTAGCCACCGCCACAAACGGCAG
PETNR_RBSOpF	GGAGGTCTTTACAGCGATGTCGGCTGAAAAACTGTTTACCCCACTGAAAGT
PETNR_Sp_RBSOpR	CCCGGTGGAGATTCCGCAACCCTGTCAATGGTGATGGTGATGGTGATGATGCAGTG
CHMO_RBSOpF	AGAGTAAAACCGACAATAGGGAGTACCGATGACCGCACAGATTAGCCCGAC
CHMO_Sp OpR	CCGGAACTGTGGGCTCCTCAACTTAGTGGTGGTGGTGGTGGTGCTC
E1C_RBSEndF	ATCTCCAGGCATCAAATAAAACGAAAGGCTCAG
E1C_StartR	TAATAACAGAGCCATATGTATATCTCCTTCTTAAAAGATCTTTTGAATTCTGAAA
Bridging oligos for the ligase cycling reaction	
L6_RBS_IPCbr1	GATCTTTTAAGAAGGAGATATACATATGGCTCTGTTATTA│GCAGTTTTTTTGTCGGCAATTATAATCCTTG TGG
L6_RBS_IPCbr2	TCCCACACTCTATAAAAGTCCGTGAGAATAACTATTTAA│GAGGGAGATTAATAACAATGGCATCTGTGAA
L6I_RBS_PCbr3	TGGCTAAATGAGAAATTCGCACTCTAGGA│GGAGGTCTTTACAGCGATGTCGGC
L6IP_SpRBS_Cbr4	ATTGACAGGGTTGCGGAATCTCCACCGGG│AGAGTAAAACCGACAATAGGGAGTACCGATGACCGCACA
L6IPC_Sp_br5	GTTGAGGAGCCCACAGTTCCGG│ATCTCCAGGCATCAAATAAAACGAAAGGC

All sequences are 5' to 3'. Bases shown underlined indicate single nucleotide mutations that generate synonymous codon mutation. The │ between bases in the bridging oligos sequences indicates the two different halves complementary to PCR DNA amplicons to be assembled next to each other.

3. Generation of L6H$_m$-IPDH-PETNR-CHMO$_{3M}$ construct version 1

Preliminary studies with the CHMO$_{WT}$ showed it reacted with (2*R*,5*R*)-dihydrocarvone to produce the abnormal lactone (3*S*,6*R*)-3-methyl-6-(prop-1-en-2-yl)oxepan-2-one.[3] However variant F249A/F280A/F435A (CHMO$_{3M}$) was shown to produce the required (+)-dihydrocarvide.[3] Therefore the wild-type CHMO was substituted for CHMO$_{3M}$ in the pBbE5c construct to generate L6HIPC$_{3M}$-E5c (Figure S2). This was performed by PCR linearisation (CHMO$_{WT}$ elimination) and amplification of L6HIPC$_{WT}$-E5c and CHMO$_{3M}$, respectively, incorporating BsrG1 and NheI restriction sites at the 5' and 3' ends, respectively. Following each PCR reaction, template removal was performed by DpnI digestion, and PCR product size was determined by 0.6% agarose gel electrophoresis. The oligonucleotide sequences encoding the PCR primers can be found in Supporting Information Table S4. Construct assembly was performed by In-Fusion cloning, and the correct assembly of each construct was confirmed by DNA sequencing. Each construct was transformed into competent cells of *E. coli* strain BL21(DE3) for functional overexpression according to the manufacturers protocols.

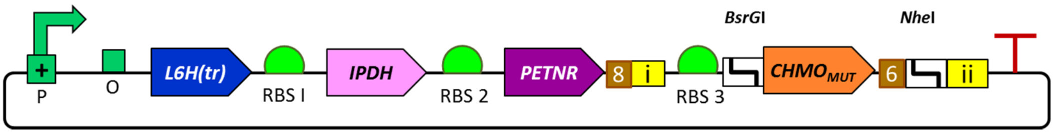

Figure S2. Design of the L6HIPC$_{3M}$-E5c construct. The yellow boxes indicate the position of the 3' spacer sequences. The IPTG inducible promoter (P) and operator (O) are shown as green arrows and boxes, respectively. The different RBS sequences are shown as green semicircles, with the terminator indicated by a red T. The numbers in brown boxes indicate the number of histidine residues in the tag. L6H(tr) = L6H$_m$.

Table S4. Oligonucleotide sequences used for the construction of L6HIPC$_{3M}$-E5c.

Name	Sequence
NheI-L-operon_F	ACCACTAAGCTAGCGTTGAGGAGCCCACAGTTC
BsrGI_L-operon_ R	TCATTGTACACGGTACTCCCTATTGTCGG
BsrGI-CHMO_F	AGTACCGTGTACAATGACCGCACAGATTAGCCCG
NheI_CHMO_R	AACGCTAGCTTAGTGGTGGTGGTGGTGGTGC

All sequences are 5' to 3'. Single and double underlined nucleotide sequence represent a BsrGI and NheI restriction sites, respectively.

4. Generation of L6H$_m$-IPDH-PETNR-CHMO$_{3M}$ constructs version 2

Additional changes were incorporated into L6HIPC$_{3M}$-E5c to see if changing the RBS upstream of CHMO$_{MUT}$ could lead to improvements in gene expression. Three variants were constructed (L6HIPC$_{3M}$-E5c 2a-2c; Figure S3) whereby three alternative RBS (RBS 3.1-3.3) were incorporated by overlap extension PCR[4] at the site of RBS 3, combined with In-Fusion cloning. Following each PCR reaction, template removal was performed by DpnI digestion, and PCR product size was determined by 0.6% agarose gel electrophoresis. The oligonucleotide sequences encoding the PCR primers can be found in Supporting Information Table S5. Construct assembly was performed by In-Fusion cloning, and the correct assembly of each construct was confirmed by DNA sequencing. Each construct was transformed into competent cells of *E. coli* strain NEB5α for functional overexpression according to the manufacturers protocols.

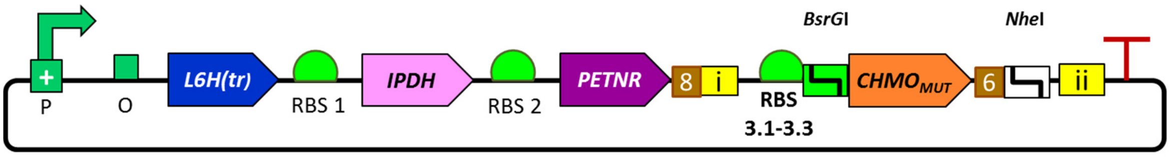

Figure S3. Design of the L6HIPC$_{3M}$ 2a-2c constructs. The yellow boxes indicate the position of the 3' spacer sequences. The IPTG inducible promoter (P) and operator (O) are shown as green arrows and boxes, respectively. The different RBS sequences are shown as green semicircles, with the terminator indicated by a red T. The numbers in brown boxes indicate the number of histidine residues in the tag. L6H(tr) = L6H$_m$.

Table S5. Oligonucleotide sequences used for the construction of L6HIPC$_{3M}$-E5c 2a-2c.

Name	Sequence
RBS3CHMO_RBS3.1_F	AGTAAGATCCCAGGGAGAGTGTACAATGACCGCACAGATTAG
RBS3CHMO_RBS3.1_R	TCCCTGGGATCTTACTTTAGCGTTGGTGGAGATTCCGCAACC
RBS3CHMO_RBS3.2_F	CCAGTAAGGAGATAATTGTACAATGACCGCACAGATTAG
RBS3CHMO_RBS3.2_R	ATTATCTCCTTACTGGGGCCTTCGTAGTGGAGATTCCGCAACC
RBS3CHMO_RBS3.3_F	CATACTAAGGAGACAAATGTACAATGACCGCACAGATTAG
RBS3CHMO_RBS3.3_R	TTGTCTCCTTAGTATGGCCTCTTGTGTGGAGATTCCGCAACC

All sequences are 5' to 3'. Underlined nucleotide sequence represents a BsrGI restriction site.

5. Generation of L6H$_{m}$-IPDH-PETNR-CHMO$_{3M}$ constructs version 3

The next stage to increasing the expression of CHMO$_{3M}$ was to incorporate a promoter and new RBS sequence upstream of the gene. The strategy employed involved PCR linearisation of construct L6HIPC$_{3M}$-E5c at the 3' end of PETNR (His$_{8}$-tag, spacer sequence and RBS3 elimination), and amplification of 4 different promoters (*trc*/*lacO*, *tacII*/*lacO*, *rha*BAD, and *lac*UV5). In-Fusion cloning between the linearised construct and each promoter-RBS generated 4 constructs (Figure S4) with CHMO$_{3M}$ controlled by different promoters, namely L6HIP-*trc*1-C$_{3M}$, L6HIP-*tac*-C$_{3M}$, L6HIP-*rha*1-C$_{3M}$ and L6HIP-*lac*UV5-C$_{3M}$. Additionally, a similar set of 3 constructs was generated, but the His$_{8}$-tag and spacer sequence after PETNR were maintained, and the chosen promoters were *trc*/*lacO*, *rha*BAD and *PtetA* (L6HIP-*trc*2-C$_{3M}$, L6HIP-*rha*2-C$_{3M}$ and L6HIP-tet-C$_{3M}$). Following each PCR reaction, template removal was performed by DpnI digestion, and PCR product size was determined by 0.6% agarose gel electrophoresis. The oligonucleotide sequences encoding the PCR primers can be found in Supporting Information Tables S6-S7. Construct assembly was performed by In-Fusion cloning, and the correct assembly of each construct was confirmed by DNA sequencing. Each construct was transformed into competent cells of *E. coli* strain NEB5α for functional overexpression according to the manufacturers protocols.

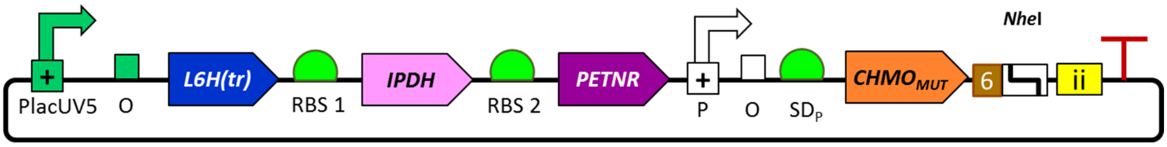

Figure S4. Design of the L6HIPpC$_{3M}$ constructs with four different promoters upstream of CHMO$_{3M}$. The yellow boxes indicate the position of the 3' spacer sequences. The IPTG inducible promoter (P) and operator (O) are shown as green arrows and boxes, respectively. SD$_{P}$ = rbs associated with the individual promoters. The different RBS sequences are shown as green semicircles, with the terminator indicated by a red T. The numbers in brown boxes indicate the number of histidine residues in the tag. L6H(tr) = L6H$_{m}$.

Table S6. Oligonucleotide sequences used for the construction of four L6HIPpC$_{3M}$ variants.

Name	Sequence
CHMO_5'_F	ATGACCGCACAGATTAGCCC
PETNR 3'_R	TCACAGTGAAGGGTAGTCGGTATAAC
CHMO_SD_LacO_F	AATGTGTGGAATTGTGAGCGGATAACAAAGGAGGATAAAGAAATGACCGCACAGATTAGCCC
PETNR_Ptrc_R*	ACAATTCCACACATTATACGAGCCGGATGATTAATTGTCAATTACAGTGAAGGGTAGTCGGTATAAC
CHMO_SD_LacO_F	AATGTGTGGAATTGTGAGCGGATAACAAAGGAGGATAAAGAAATGACCGCACAGATTAGCCC
PETNR_PtacII-R	ACAATTCCACACATTAAACTAGTTCGATGATTAATTGTCAATTACAGTGAAGGGTAGTCGGTATAAC
BiTerm-PETNR_F	TTATACCGACTACCCTTCACTGTGAAAAGTCAAAAGCCTCCGACCG
PrhaB-SD-CHMOm_R	TAATCTGTGCGGTCATTTCTTTATCCTCCTTTCATTACGACCAGTCTAAAAAGCG
LacUV5_PETNR_F	TACCCTTCACTGTGATAGGCACCCCAGGCTTTAC
LacO_SD_CHMO_R	AATCTGTGCGGTCATTTCTTTATcctcctTTGTTATCCGCTCACAATTCCAC

All sequences are 5' to 3'. Underlined nucleotide sequence represents a BsrGI restriction site.

Table S7. Oligonucleotide sequences used for the construction of four L6HIPpC$_{3M}$ variants.

Name	Sequence
CHMO_5'_F	ATGACCGCACAGATTAGCCC
Spacer-R	TGGAGATTCCGCAACCCTG
CHMO_SD_LacO_F	AATGTGTGGAATTGTGAGCGGATAACAAAGGAGGATAAAGAAATGACCGCACAGATTAGCCC
Spacer_Ptrc_LacO_R	CGCTCACAATTCCACACATTATACGAGCCGGATGATTAATTGTCAAGTGGAGATTCCGCAACCCT
Biterm-Spacer*F	GGGTTGCGGAATCTCCACAAAGTCAAAAGCCTCCGACCG
PrhaB-SD-CHMOm*R	GGCTAATCTGTGCGGTCATTTCTTTATCCTCCTTTCATTACGACCAGTCTAAAAAGCG
TetR-Spacer*F	GGGTTGCGGAATCTCCACTTAAGACCCACTTTCACATTTAAGT
Ptet-SD-CHMO*R	GGCTAATCTGTGCGGTCATTTCTTTATCCTCCTTTCTCTATCACTGATAGGGAGTGG
LacUV5_PETNR_F	TACCCTTCACTGTGATAGGCACCCCAGGCTTTAC
LacO_SD_CHMO_R	AATCTGTGCGGTCATTTCTTTATcctcctTTGTTATCCGCTCACAATTCCAC

All sequences are 5' to 3'. Underlined nucleotide sequence represents a BsrGI restriction site.

6. *In vitro* biotransformation of L6H$_m$-IPDH-PETNR-CHMO$_{3M}$ constructs

A single colony of *E. coli* BL21(DE3) containing an L6H$_m$-IPDH-PETNR-CHMO$_{3M}$ construct was used to inoculate 5 mL of LB medium, containing 34 μg/mL chloramphenicol. The culture was incubated at 37°C, 200 rpm until reaching OD600 = 0.4, followed by recombinant protein induction by 100 μM IPTG (*trc*/*lacO*, *tacII*/*lacO* and *lac*UV5 promoters). For constructs containing a second *rha*BAD or *PtetA* promoter upstream of CHMO$_{3M}$, 0.05% rhamnose or 100 nM tetracycline was added with the IPTG, respectively. Cultures were incubated at 30 °C for 72 hours unless otherwise stated. Cells were harvested by centrifugation (4000 x *g*) and the pellets were resuspended in 1.7 mL lysis buffer (50 mM Tris pH 7.0 containing the EDTA-free complete protease inhibitor cocktail, 1 mM $MgCl_2$, 0.1 mg/mL DNase I, 0.1 mg/mL lysozyme and 10% glycerol). Cell-free supernatants were generated by sonication (10 cycles of 10 s on/1 min off at 40 % amplitude).

Reactions (1 mL) were performed in buffer (50 mM Tris pH 7.0) containing cell lysate, 5 mM (-)-carveol isomer mix, 15 μM $NADP^+$, 150 μM NAD^+, 15 mM glucose and 10 U GDH. Reactions were incubated at 30 °C for 24 h at 180 rpm. Control reactions were performed with *E. coli* lysates that did not contain the recombinant plasmids. Monoterpenoids were extracted with 2 x 0.5 mL ethyl acetate containing 0.1 % *sec*-butylbenzene internal standard. Product yields and identification were determined by GCMS analysis, using a DB-WAX column.

7. Generation of a full lactone-producing pathway

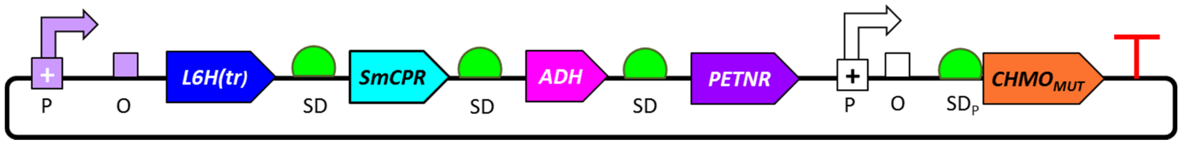

Figure S5. Design of the three L6HCCPpC3M constructs in pBbB8k-vector with different promoters upstream of CHMO3M. The different RBS sequences are shown as green semicircles, with the terminator indicated by a red T. L6H(tr) = L6Hm.

Table S8. Oligonucleotide sequences used for the construction of three L6HCCPpC3M variants.

Name	Sequence
pBbB8k_rrnB*_F	GGATCTCCAGGCATCAAATAAAACG
CDH_RBS2mut_R	CCTCCTAGAGTGCGAATTTCTCATTTCAGGGTAGTGCCAGCG
CDH-RB2_F	CCTCCTAGAGTGCGAATTTCTCATTTCAGGGTCGTGCCA
pBbB8k_rrnB_R	GGATCTCCAGGCATCAAATAAA
CDH-RB2_F	CCTCCTAGAGTGCGAATTTCTCATTTCAGGGTCGTGCCA
pBbB8k_rrnB_R	GGATCTCCAGGCATCAAATAAA
CDH-RB2_F	CCTCCTAGAGTGCGAATTTCTCATTTCAGGGTCGTGCCA
pBbB8k_rrnB_R	GGATCTCCAGGCATCAAATAAA

All sequences are 5’ to 3’.

8. Summary of constructs

Table S9. Multi gene constructs generated in this project.

Construct	Order of genes	Promoter(s)	Vector(s)[a]	Other
L6H$_m$-SmCPR	L6H$_m$ SmCPR	*lac*UV5	pCWori (+)	-
		*ara*BAD	pBbB8k	-
		tetA	pBbE2k	-
L6H$_m$-AtCPR	L6H$_m$ AtCPR	*lac*UV5	pCWori (+)	-
		*ara*BAD	pBbB8k	-
		tetA	pBbE2k	-
L6H$_m$-SmCPR-IPDH v1	L6H$_m$ SmCPR IPDH	*ara*BAD	pBbB8k	RBS 1
L6H$_m$-SmCPR-IPDH v2	L6H$_m$ SmCPR IPDH	*ara*BAD	pBbB8k	RBS 2
L6H$_m$-SmCPR-CDH v1	L6H$_m$ SmCPR CDH	*ara*BAD	pBbB8k	RBS 1
L6H$_m$-SmCPR-CDH v2	L6H$_m$ SmCPR CDH	*ara*BAD	pBbB8k	RBS 2
L6H$_m$-SmCPR-LkADH v1	L6H$_m$ SmCPR LkADH	*ara*BAD	pBbB8k	RBS 1
L6H$_m$-SmCPR-LkADH v2	L6H$_m$ SmCPR LkADH	*ara*BAD	pBbB8k	RBS 2
L6H$_m$-SmCPR-RRADH v1	L6H$_m$ SmCPR RRADH	*ara*BAD	pBbB8k	RBS 1
L6H$_m$-SmCPR-RRADH v2	L6H$_m$ SmCPR RRADH	*ara*BAD	pBbB8k	RBS 2
L6HIPC$_{WT}$	L6H$_m$ IPDH PETNR CHMO$_{WT}$	*trc*/*lacO*	pBbE1c	-
		*lac*UV5	pBbE5c	-
L6HIPC$_{3M}$-E5c	L6H$_m$ IPDH PETNR CHMO$_{3M}$	*lac*UV5	pBbE5c	RBS 3.0
L6HIPC$_{3M}$-E5c 2a	L6H$_m$ IPDH PETNR CHMO$_{3M}$	*lac*UV5	pBbE5c	RBS 3.1
L6HIPC$_{3M}$-E5c 2b	L6H$_m$ IPDH PETNR CHMO$_{3M}$	*lac*UV5	pBbE5c	RBS 3.2
L6HIPC$_{3M}$-E5c 2c	L6H$_m$ IPDH PETNR CHMO$_{3M}$	*lac*UV5	pBbE5c	RBS 3.3
L6HIP-*trc*1-C$_{3M}$	L6H$_m$ IPDH PETNR CHMO$_{3M}$	*lac*UV5 and *trc*/*lacO*	pBbE5c	-
L6HIP-*tac*-C$_{3M}$	L6H$_m$ IPDH PETNR CHMO$_{3M}$	*lac*UV5 and *tacII*/*lacO*	pBbE5c	-
L6HIP-*rha*1-C$_{3M}$	L6H$_m$ IPDH PETNR CHMO$_{3M}$	*lac*UV5 and *rha*BAD	pBbE5c	-
L6HIP-*lac*UV5-C$_{3M}$	L6H$_m$ IPDH PETNR CHMO$_{3M}$	*lac*UV5 and *lac*UV5	pBbE5c	-
L6HIP-*trc*2-C$_{3M}$	L6H$_m$ IPDH PETNR CHMO$_{3M}$	*lac*UV5 and *trc*/*lacO*	pBbE5c	-
L6HIP-*rha*2-C$_{3M}$	L6H$_m$ IPDH PETNR CHMO$_{3M}$	*lac*UV5 and *ara*BAD	pBbE5c	-
L6HIP-*tet*-C$_{3M}$	L6H$_m$ IPDH PETNR CHMO$_{3M}$	*lac*UV5 and *tetA*	pBbE5c	-
L6HCCP-*tet*-C$_{3M}$	L6H$_m$ SmCPR CDH PETNR CHMO$_{3M}$	*ara*BAD and *tetA*	pBbB8k	-
L6HCCP-*rha*-C$_{3M}$	L6H$_m$ SmCPR CDH PETNR CHMO$_{3M}$	*ara*BAD and *ara*BAD	pBbB8k	-
L6HCCP-*trc*-C$_{3M}$	L6H$_m$ SmCPR CDH PETNR CHMO$_{3M}$	*ara*BAD and *trc*/*lacO*	pBbB8k	-

[a] Second promoter is upstream of the CHMO$_{3M}$ gene.

Results and Discussion

1. *In vitro* biotransformations of cell extracts of L6H$_m$ co-expressed with SmCPR or AtCPR

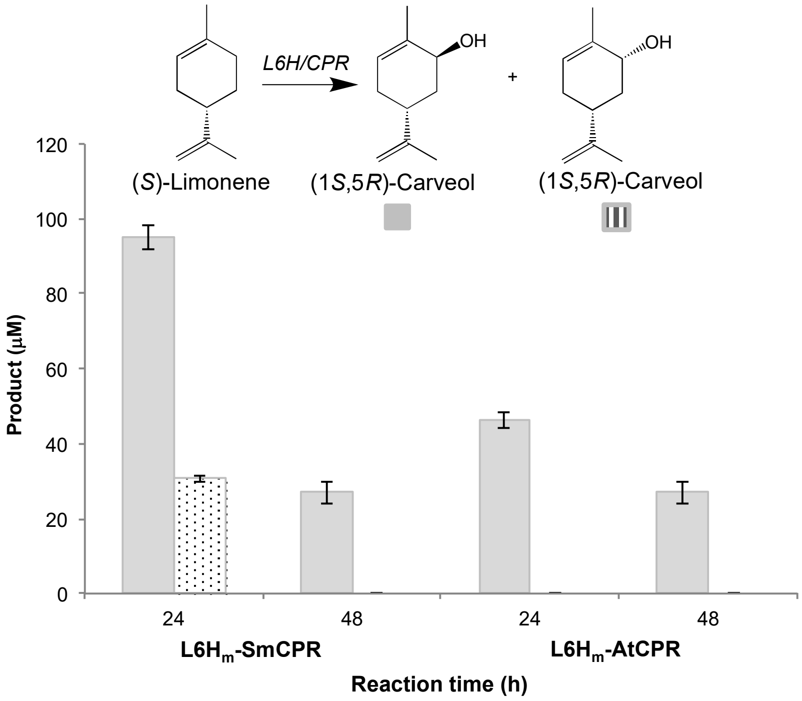

Figure S6. *In vitro* monoterpenoid production by constructs L6H$_m$-SmCPR and L6H$_m$-AtCPR in pCWori. Reactions (1 mL) were performed in buffer (50 mM Tris pH 7.0) containing cell lysate, 5 mM limonene, 150 µM NAD$^+$ $\pm$ 15 µM NADP$^+$, 15 mM glucose and 10 U GDH. Reactions were incubated at 30 °C for 24 h at 180 rpm. Monoterpenoids were extracted with 2 x 0.5 mL ethyl acetate containing 0.1 % *sec*-butylbenzene internal standard. Product yields and identification were determined by GCMS analysis, using a DB-WAX column. Data is the average of duplicate reactions, and the error is 1 standard deviation of the data.

2. *In vivo* biotransformations of the L6H$_M$-SmCPR construct

Table S10. *In vivo* (1*S*,5*R*)-carveol production by L6H$_m$-SmCPR in pBbB8k.

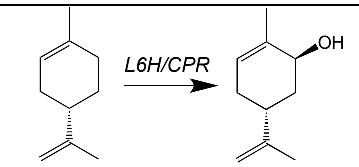

(*S*)-Limonene (1*S*,5*R*)-Carveol

Kanamycin (μg/mL)	Nonane (Y/N)	Induction (OD_{600})	IPTG (μM)	Arabinose (mM)	(1*S*,5*R*)-Carveol (mg/L/OD_{600})
Effect of kanamycin concentration					
15	Yes	0.4	25	10	1.7 ± 0.9
15	No	0.4	25	10	12.8 ± 4.4
60	Yes	0.4	25	10	3.1 ± 2.5
60	No	0.4	25	10	40.9 ± 18.8
Effect of induction OD_{600}					
60	No	0.01	25	10	4.65 ± 2.29
60	No	0.025	25	10	4.78 ± 2.41
60	No	0.05	25	10	7.51 ± 2.94
60	No	0.1	25	10	15.35 ± 0.71
60	No	0.2	25	10	25.37 ± 6.96
60	No	0.4	25	10	28.33 ± 6.63
60	No	0.8	25	10	14.61 ± 3.28
Effect of inducer concentration					
60	No	0.4	5	10	1.61 ± 0.17
60	No	0.4	25	10	6.74 ± 0.20
60	No	0.4	100	10	1.95 ± 0.35
60	No	0.4	500	10	2.30 ± 0.55
60	No	0.4	25	2	13.99 ± 5.88
60	No	0.4	25	10	24.50 ± 2.63
60	No	0.4	25	25	33.78 ± 4.99
60	No	0.4	25	100	32.71 ± 7.70

Reactions (5 mL) were performed in Terrific Broth buffer containing phosphate salt (9.4 g/L KH_2PO4 and 2.2 g/LK_2PO_4) and 0.7 %(w/v) glucose, kanamycin and ampicillin (100 µg/mL). Reactions were incubated for 37 °C and 200 rpm until reaching OD600 = 0.4, followed by supplementation of IPTG, arabinose and δ-aminolevulinic acid (500 nM). The cultures were incubated at 30 °C for 72 hours unless and each culture aliquot (3 mL) was cooled for 10 minutes on ice. Monoterpenoids were extracted with 2 x 0.375 mL ethyl acetate containing 0.01 % *sec*-butylbenzene internal standard. Product yields and identification were determined by GCMS analysis, using a DB-WAX column. Data is the average of triplicate reactions, and the error is 1 standard deviation of the data.

3. *In vitro* biotransformations of the L6HIPC3M-E5c construct

Table S11. *In vitro* monoterpenoid production by construct L6HIPC3M-E5c.

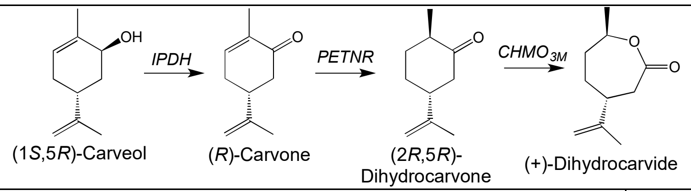

		Monoterpenoid product (mM)				By-products (mM)[1]	
Substrate	Cofactors	(*R*)-Carvone	(2*R*,5*R*)-DHC	(2*S*,5*R*)-DHC	(+)-DHCD	(1*S*,2*R*,5*R*)-DHCL	Carvyl Ac.
Carveol	$NADP^+/NAD^+$	0.87 ± 0.03	0.34 ± 0.03	0.02 ± 0.01	ND	1.04 ± 0.19	0.38 ± 0.07
	NAD^+	2.64 ± 0.24	0.16 ± 0.02	0.03 ± 0.02	ND	ND	0.56 ± 0.10
Carvone	$NADP^+/NAD^+$	-	1.47 ± 0.08	0.23 ± 0.01	0.11 ± 0.01	0.25 ± 0.05	ND
	NAD^+	-	1.02 ± 0.11	0.24 ± 0.02	0.19 ± 0.03	0.05 ± 0.01	ND
DHC	$NADP^+/NAD^+$	-	-	-	ND	0.26 ± 0.05	ND
	NAD^+	-	-	-	0.05 ± 0.01	0.18 ± 0.03	ND

Reactions (1 mL) were performed in buffer (50 mM Tris pH 7.0) containing cell lysate, 5 mM monoterpenoid, 150 μM NAD^+ ± 15 μM $NADP^+$, 15 mM glucose and 10 U GDH. Reactions were incubated for 30 °C for 24 h at 180 rpm. Monoterpenoids were extracted with 2 x 0.5 mL ethyl acetate containing 0.1 % *sec*-butylbenzene internal standard. Product yields and identification were determined by GCMS analysis, using a DB-WAX column. Data is the average of triplicate reactions, and the error is 1 standard deviation of the data. [1]By-product concentrations are estimations as no commercially available standards were available. Substrates: Carveol = (1*S*,5*R*)- and (1*R*,5*R*)-carveol isomer mix; carvone = (*R*)-carvone; DHC = (2*R*,5*R*)- and (2*S*,5*R*)-dihydrocarvone isomer mix. Products: DHC = dihydrocarvone; DHCD = dihydrocarvide lactone; DHCL = dihydrocarveol, Carvyl Ac. = carvyl acetate. ND = none detected.

Enzymes L6H/CPR, PETNR and CHMO3M prefer NADPH for activity, while IPDH is NAD^+ dependent. Therefore reactions in the presence of only exogenously added NAD^+ utilise $NADP^+$ present in the cell extracts. The highest yields of (+)-dihydrocarvide were detected when (*R*)-carvone was the substrate (PETNR and CHMO3M activity only). Significant quantities of by-products were detected, such as carvyl acetate. This is likely generated by the acetylation of (-)-carveol by chloramphenicol acetyltransferase.[5] The production of dihydrocarveol is likey due to ADH activity on dihydrocarvone.

4. *In vitro* biotransformations of the L6HIPC$_{3M}$ 2a-2c constructs

Table S12. *In vitro* monoterpenoid production by constructs L6HIPC$_{3M}$-E5c 2a-2c (RBS 3.1-3.3).

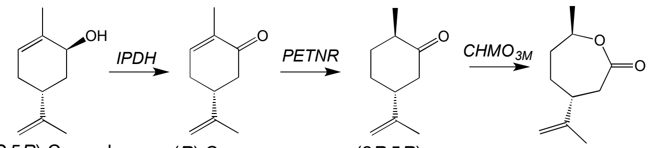

(1*S*,5*R*)-Carveol (*R*)-Carvone (2*R*,5*R*)-Dihydrocarvone (+)-Dihydrocarvide

		Monoterpenoid product (mM)				By-products (mM)[1]	
Substrate	Cofactors	(*R*)-Carvone	(2*R*,5*R*)-DHC	(2*S*,5*R*)-DHC	(+)-DHCD	(1*S*,2*R*,5*R*)-DHCL	Carvyl Ac.
RBS 3.1							
Carveol	$NADP^+/NAD^+$	0.09 ± 0.01	0.12 ± 0.01	0.02 ± 0.01	ND	0.24 ± 0.04	0.38 ± 0.07
	NAD^+	0.16 ± 0.03	0.03 ± 0.01	ND	ND	0.31 ± 0.06	0.56 ± 0.10
Carvone	$NADP^+/NAD^+$	-	2.03 ± 0.08	0.44 ± 0.01	ND	ND	ND
	NAD^+	-	0.70 ± 0.25	0.20 ± 0.01	ND	0.04 ± 0.01	ND
DHC	$NADP^+/NAD^+$	-	-	-	ND	0.07 ± 0.01	ND
	NAD^+	-	-	-	ND	0.07 ± 0.01	ND
RBS 3.2							
Carveol	$NADP^+/NAD^+$	0.11 ± 0.01	0.10 ± 0.01	0.01 ± 0.01	ND	0.42 ± 0.08	0.38 ± 0.07
	NAD^+	0.24 ± 0.01	0.05 ± 0.01	ND	ND	0.44 ± 0.08	0.56 ± 0.10
Carvone	$NADP^+/NAD^+$	-	2.47 ± 0.36	0.54 ± 0.06	ND	ND	ND
	NAD^+	-	1.39 ± 0.05	0.32 ± 0.02	ND	0.08 ± 0.01	ND
DHC	$NADP^+/NAD^+$	-	-	-	ND	0.18 ± 0.03	ND
	NAD^+	-	-	-	ND	0.08 ± 0.01	ND
RBS 3.3							
Carveol	$NADP^+/NAD^+$	0.06 ± 0.03	1.16 ± 1.04	0.26 ± 0.24	ND	0.24 ± 0.04	0.38 ± 0.07
	NAD^+	0.17 ± 0.02	0.05 ± 0.01	ND	ND	0.21 ± 0.03	0.56 ± 0.10
Carvone	$NADP^+/NAD^+$	-	2.62 ± 0.13	0.52 ± 0.04	ND	0.06 ± 0.01	ND
	NAD^+	-	1.03 ± 0.14	0.22 ± 0.04	ND	0.05 ± 0.01	ND
DHC	$NADP^+/NAD^+$	-	-	-	ND	0.10 ± 0.02	ND
	NAD^+	-	-	-	ND	0.09 ± 0.02	ND

Reactions (1 mL) were performed in buffer (50 mM Tris pH 7.0) containing cell lysate, 5 mM monoterpenoid, 150 μM NAD^+ ± 15 μM $NADP^+$, 15 mM glucose and 10 U GDH. Reactions were incubated for 30 °C for 24 h at 180 rpm. Monoterpenoids were extracted with 2 x 0.5 mL ethyl acetate containing 0.1 % *sec*-butylbenzene internal standard. Product yields and identification were determined by GCMS analysis, using a DB-WAX column. Data is the average of triplicate reactions, and the error is 1 standard deviation of the data. [1]By-product concentrations are estimations as no commercially available standards were available. Substrates: Carveol = (1*S*,5*R*)- and (1*R*,5*R*)-carveol isomer mix; carvone = (*R*)-carvone; DHC = (2*R*,5*R*)- and (2*S*,5*R*)-dihydrocarvone isomer mix. Products: DHC = dihydrocarvone; DHCD = dihydrocarvide lactone; DHCL = dihydrocarveol, Carvyl Ac. = carvyl acetate. ND = none detected.

Unlike the L6HIPC$_{3M}$-E5c construct, no (+)-dihydrocarvide was detected in the reactions. As these constructs differ only by the sequence of the RBS upstream of CHMO$_{3M}$, it is likely that the loss of activity is due to lower enzyme expression. As expected, higher levels of dihydrocarvone isomers were detected when $NADP^+/NAD^+$ were used, as PETNR prefers NADPH over NADH.

5. *In vitro* biotransformations of the seven L6HIPpC$_{3M}$ constructs

Table S13. *In vitro* monoterpenoid production by constructs L6HIPpC$_{3M}$-trc-1, L6HIPpC$_{3M}$-tac, L6HIPpC$_{3M}$-pRhaBAD-1 and L6HIPpC$_{3M}$-UV5.

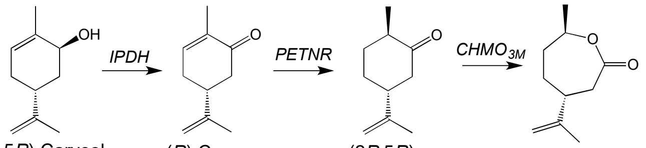

(1*S*,5*R*)-Carveol (*R*)-Carvone (2*R*,5*R*)-Dihydrocarvone (+)-Dihydrocarvide

		Monoterpenoid product (mM)				By-products (mM)[1]	
Substrate	Cofactors	(*R*)-Carvone	(2*R*,5*R*)-DHC	(2*S*,5*R*)-DHC	(+)-DHCD	(1*S*,2*R*,5*R*)-DHCL	Carvyl Ac.
Construct L6HIP-trc1-C$_{3M}$							
Carveol	NADP$^+$/NAD$^+$	0.04 ± 0.01	0.05 ± 0.01	ND	0.12 ± 0.01	0.04 ± 0.01	0.29 ± 0.05
	NAD$^+$	0.08 ± 0.01	0.09 ± 0.01	0.01 ± 0.01	ND	0.22 ± 0.04	0.96 ± 0.17
Carvone	NADP$^+$/NAD$^+$	-	2.36 ± 0.13	0.51 ± 0.03	0.47 ± 0.06	0.01 ± 0.01	ND
	NAD$^+$	-	0.69 ± 0.03	0.15 ± 0.01	0.10 ± 0.01	ND	ND
DHC	NADP$^+$/NAD$^+$	-	-	-	0.57 ± 0.07	0.02 ± 0.01	ND
	NAD$^+$	-	-	-	0.07 ± 0.01	0.02 ± 0.01	ND
Construct L6HIP-tac-C$_{3M}$							
Carveol	NADP$^+$/NAD$^+$	0.14 ± 0.01	0.11 ± 0.01	0.02 ± 0.01	ND	0.27 ± 0.05	0.80 ± 0.14
	NAD$^+$	0.28 ± 0.01	0.04 ± 0.01	ND	ND	0.22 ± 0.04	0.96 ± 0.17
Carvone	NADP$^+$/NAD$^+$	-	2.23 ± 0.09	0.52 ± 0.02	0.38 ± 0.10	0.05 ± 0.01	ND
	NAD$^+$	-	0.64 ± 0.07	0.15 ± 0.02	0.17 ± 0.01	0.03 ± 0.01	ND
DHC	NADP$^+$/NAD$^+$	-	-	-	0.32 ± 0.04	0.06 ± 0.01	ND
	NAD$^+$	-	-	-	0.16 ± 0.04	ND	ND
Construct L6HIP-rha1-C$_{3M}$							
Carveol	NADP$^+$/NAD$^+$	0.16 ± 0.03	0.14 ± 0.01	0.03 ± 0.01	ND	0.43 ± 0.08	0.76 ± 0.14
	NAD$^+$	0.38 ± 0.01	0.04 ± 0.01	ND	ND	0.32 ± 0.06	0.93 ± 0.17
Carvone	NADP$^+$/NAD$^+$	-	2.21 ± 0.03	0.51 ± 0.01	0.35 ± 0.06	0.09 ± 0.02	ND
	NAD$^+$	-	0.95 ± 0.08	0.22 ± 0.02	0.27 ± 0.04	0.11 ± 0.02	ND
DHC	NADP$^+$/NAD$^+$	-	-	-	0.30 ± 0.02	0.08 ± 0.01	ND
	NAD$^+$	-	-	-	0.14 ± 0.03	0.08 ± 0.01	ND
Construct L6HIP-lacUV5-C$_{3M}$							
Carveol	NADP$^+$/NAD$^+$	0.11 ± 0.01	0.13 ± 0.01	0.02 ± 0.01	ND	0.27 ± 0.05	0.54 ± 0.10
	NAD$^+$	0.20 ± 0.02	0.07 ± 0.01	0.01 ± 0.01	ND	0.19 ± 0.03	0.65 ± 0.12
Carvone	NADP$^+$/NAD$^+$	-	2.68 ± 0.06	0.60 ± 0.02	0.28 ± 0.06	0.05 ± 0.01	ND
	NAD$^+$	-	0.94 ± 0.05	0.22 ± 0.01	0.25 ± 0.07	0.04 ± 0.01	ND
DHC	NADP$^+$/NAD$^+$	-	-	-	0.30 ± 0.04	0.08 ± 0.01	ND
	NAD$^+$	-	-	-	0.12 ± 0.03	0.07 ± 0.01	ND

Reactions (1 mL) were performed in buffer (50 mM Tris pH 7.0) containing cell lysate, 5 mM monoterpenoid, 150 μM NAD$^+$ ± 15 μM NADP$^+$, 15 mM glucose and 10 U GDH. Reactions were incubated at 30 °C for 24 h at 180 rpm. Monoterpenoids were extracted with 2 x 0.5 mL ethyl acetate containing 0.1 % *sec*-butylbenzene internal standard. Product yields and identification were determined by GCMS analysis, using a DB-WAX column. Data is the average of triplicate reactions, and the error is 1 standard deviation of the data. [1]By-product concentrations are estimations as no commercially available standards were available. Substrates: Carveol = (1*S*,5*R*)- and (1*R*,5*R*)-carveol isomer mix; carvone = (*R*)-carvone; DHC = (2*R*,5*R*)- and (2*S*,5*R*)-dihydrocarvone isomer mix. Products: DHC = dihydrocarvone; DHCD = dihydrocarvide lactone; DHCL = dihydrocarveol, Carvyl Ac. = carvyl acetate. ND = none detected.

Each construct generated dihydrocarvide lactone, but only the pTrc-1 construct showed this activity starting from carveol. Reactions containing cofactor combinations $NADP^+$/NAD^+ generated higher lactone than reactions containing NAD^+ alone.

Table S14. *In vitro* monoterpenoid production by constructs L6HIPpC3M-trc-2, L6HIPpC3M-pBAD-2 and L6HIPpC3M-pTet.

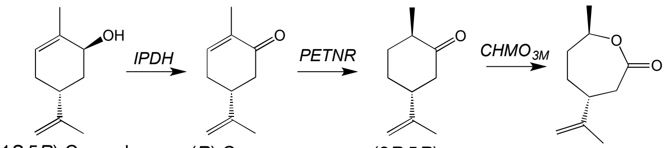

(1*S*,5*R*)-Carveol (*R*)-Carvone (2*R*,5*R*)-Dihydrocarvone (+)-Dihydrocarvide

		Monoterpenoid product (mM)				By-products (mM)[1]	
Substrate	Cofactors	(*R*)-Carvone	(2*R*,5*R*)-DHC	(2*S*,5*R*)-DHC	(+)-DHCD	(1*S*,2*R*,5*R*)-DHCL	Carvyl Ac.
Construct L6HIP-trc2-C3M							
Carveol	NADP+/NAD+	0.02 ± 0.01	0.07 ± 0.01	ND	0.13 ± 0.01	0.03 ± 0.01	1.60 ± 0.11
	NAD+	0.11 ± 0.01	0.05 ± 0.01	ND	ND	ND	0.14 ± 0.02
Carvone	NADP+/NAD+	-	1.18 ± 0.05	0.02 ± 0.01	0.61 ± 0.10	ND	ND
	NAD+	-	0.18 ± 0.01	ND	ND	ND	ND
DHC	NADP+/NAD+	-	-	-	0.77 ± 0.03	ND	ND
	NAD+	-	-	-	ND	ND	ND
Construct L6HIP-rha2-C3M							
Carveol	NADP+/NAD+	0.08 ± 0.01	0.15 ± 0.01	ND	ND	0.41 ± 0.07	0.34 ± 0.06
	NAD+	0.23 ± 0.02	0.07 ± 0.01	ND	ND	0.26 ± 0.05	0.27 ± 0.05
Carvone	NADP+/NAD+	-	2.29 ± 0.07	ND	0.11 ± 0.02	0.10 ± 0.02	ND
	NAD+	-	0.65 ± 0.08	0.01 ± 0.01	ND	ND	ND
DHC	NADP+/NAD+	-	-	-	0.13 ± 0.03	ND	ND
	NAD+	-	-	-	ND	ND	ND
Construct L6HIP-tet-C3M-							
Carveol	NADP+/NAD+	0.02 ± 0.01	0.11 ± 0.01	ND	0.05 ± 0.02	0.03 ± 0.01	0.18 ± 0.03
	NAD+	0.15 ± 0.01	0.06 ± 0.01	ND	ND	0.02 ± 0.01	0.13 ± 0.02
Carvone	NADP+/NAD+	-	0.92 ± 0.15	0.02 ± 0.01	0.28 ± 0.05	ND	ND
	NAD+	-	0.13 ± 0.01	ND	ND	ND	ND
DHC	NADP+/NAD+	-	-	-	0.33 ± 0.06	ND	ND
	NAD+	-	-	-	ND	ND	ND

Reactions (1 mL) were performed in buffer (50 mM Tris pH 7.0) containing cell lysate, 5 mM monoterpenoid, 150 μM NAD^+ ± 15 μM $NADP^+$, 15 mM glucose and 10 U GDH. Reactions were incubated at 30 °C for 24 h at 180 rpm. Monoterpenoids were extracted with 2 x 0.5 mL ethyl acetate containing 0.1 % *sec*-butylbenzene internal standard. Product yields and identification were determined by GCMS analysis, using a DB-WAX column. Data is the average of triplicate reactions, and the error is 1 standard deviation of the data. [1]By-product concentrations are estimations as no commercially available standards were available. Substrates: Carveol = (1*S*,5*R*)- and (1*R*,5*R*)-carveol isomer mix; carvone = (*R*)-carvone; DHC = (2*R*,5*R*)- and (2*S*,5*R*)-dihydrocarvone isomer mix. Products: DHC = dihydrocarvone; DHCD = dihydrocarvide lactone; DHCL = dihydrocarveol, Carvyl Ac. = carvyl acetate. ND = none detected.

The highest yields of (+)-dihydrocarvide were detected in the construct containing pTrc promoter upstream of CHMO3M. As expected, lower yields of (+)-dihydrocarvide were obtained when p*rha*BAD was the promoter, as this is known to have a lower expression strength than IPTG-based promoters.

References

[1] T. S. Lee, R. A. Krupta, F. Zhang, M. Hajimorad, W. J. Holtz, N. Prasad, S. K. Lee, J. D. Keasling, *J. Biol. Eng.* **2011**, *5*, 12.

[2] S. de Kok, L. H. Stanton, T. Slaby, M. Durot, V. F. Holmes, K. G. Patel, D. Platt, E. B. Shapland, Z. Serber, J. Dean, J. D. Newman, S. S. Chandran, *ACS Synthetic Biology* **2014**, *3*, 97-106.

[3] H. L. Messiha, S. T. Ahmed, V. Karuppiah, R. Suardiaz, G. A. Ascue Avalos, N. Fey, S. Yeates, H. S. Toogood, A. J. Mulholland, N. S. Scrutton, *Biochemistry* **2018**, *57*, 1997-2008.

[4] Y.-H. Xiao, M.-H. Yin, L. Hou, M. Luo, Y. Pei, *Biotechnol. Lett.* **2007**, *29*, 925-930.

[5] J. Alonso-Gutierrez, R. Chan, T. S. Batth, P. D. Adams, J. D. Keasling, C. J. Petzold, T. S. Lee, *Metabolic Engineering* **2013**, *19*, 33-41.
